# Molecular modulators of cyclin-dependent kinase 4/6 inhibitor response in experimental glioma identified through genome-wide CRISPR-Cas9 screening

**DOI:** 10.1093/neuonc/noag093

**Published:** 2026-05-04

**Authors:** Surender Surender, Lara Annina Haeusser, Laurence Kuhlburger, Foteini Tsiami, Nihal Olcay Dogan, Louise Maise, Sven Nahnsen, Susanne Beck, Daniel Josef Merk, Ghazaleh Tabatabai

**Affiliations:** Department of Neurology and Interdisciplinary Neuro-Oncology, Hertie Institute for Clinical Brain Research, University Hospital Tübingen, Eberhard Karls University, Tübingen, Germany; Cluster of Excellence iFIT (EXC 2180) “Image Guided and Functionally Instructed Tumor Therapies”, Eberhard Karls University, Tübingen, Germany; German Cancer Consortium (DKTK), Partner Site Tübingen, A Partnership Between DKFZ and University Hospital Tübingen, Tübingen, Germany; Department of Neurology and Interdisciplinary Neuro-Oncology, Hertie Institute for Clinical Brain Research, University Hospital Tübingen, Eberhard Karls University, Tübingen, Germany; Cluster of Excellence iFIT (EXC 2180) “Image Guided and Functionally Instructed Tumor Therapies”, Eberhard Karls University, Tübingen, Germany; German Cancer Consortium (DKTK), Partner Site Tübingen, A Partnership Between DKFZ and University Hospital Tübingen, Tübingen, Germany; Department of Neurology and Interdisciplinary Neuro-Oncology, Hertie Institute for Clinical Brain Research, University Hospital Tübingen, Eberhard Karls University, Tübingen, Germany; Quantitative Biology Center, Eberhard Karls University Tübingen, Tübingen, Germany; Biomedical Data Science, Department of Computer Science, Eberhard Karls University Tübingen, Tübingen, Germany; Department of Neurology and Interdisciplinary Neuro-Oncology, Hertie Institute for Clinical Brain Research, University Hospital Tübingen, Eberhard Karls University, Tübingen, Germany; Cluster of Excellence iFIT (EXC 2180) “Image Guided and Functionally Instructed Tumor Therapies”, Eberhard Karls University, Tübingen, Germany; Department of Neurology and Interdisciplinary Neuro-Oncology, Hertie Institute for Clinical Brain Research, University Hospital Tübingen, Eberhard Karls University, Tübingen, Germany; Cluster of Excellence iFIT (EXC 2180) “Image Guided and Functionally Instructed Tumor Therapies”, Eberhard Karls University, Tübingen, Germany; Department of Neurology and Interdisciplinary Neuro-Oncology, Hertie Institute for Clinical Brain Research, University Hospital Tübingen, Eberhard Karls University, Tübingen, Germany; Cluster of Excellence iFIT (EXC 2180) “Image Guided and Functionally Instructed Tumor Therapies”, Eberhard Karls University, Tübingen, Germany; Cluster of Excellence iFIT (EXC 2180) “Image Guided and Functionally Instructed Tumor Therapies”, Eberhard Karls University, Tübingen, Germany; Quantitative Biology Center, Eberhard Karls University Tübingen, Tübingen, Germany; Biomedical Data Science, Department of Computer Science, Eberhard Karls University Tübingen, Tübingen, Germany; Department of Neurology and Interdisciplinary Neuro-Oncology, Hertie Institute for Clinical Brain Research, University Hospital Tübingen, Eberhard Karls University, Tübingen, Germany; Cluster of Excellence iFIT (EXC 2180) “Image Guided and Functionally Instructed Tumor Therapies”, Eberhard Karls University, Tübingen, Germany; Department of Neurology and Interdisciplinary Neuro-Oncology, Hertie Institute for Clinical Brain Research, University Hospital Tübingen, Eberhard Karls University, Tübingen, Germany; Cluster of Excellence iFIT (EXC 2180) “Image Guided and Functionally Instructed Tumor Therapies”, Eberhard Karls University, Tübingen, Germany; Department of Neurology and Interdisciplinary Neuro-Oncology, Hertie Institute for Clinical Brain Research, University Hospital Tübingen, Eberhard Karls University, Tübingen, Germany; Cluster of Excellence iFIT (EXC 2180) “Image Guided and Functionally Instructed Tumor Therapies”, Eberhard Karls University, Tübingen, Germany; German Cancer Consortium (DKTK), Partner Site Tübingen, A Partnership Between DKFZ and University Hospital Tübingen, Tübingen, Germany; Center for Neuro-Oncology, Comprehensive Cancer Center Tübingen Stuttgart, Eberhard Karls University, Tübingen, Germany

## Abstract

**Background:**

Glioblastoma harbors frequent alterations in the retinoblastoma pathway, providing a genetic rationale for therapeutic targeting with cyclin-dependent kinase 4/6 (CDK4/6) inhibitors. The NOA-20 trial did not reveal a progression-free survival benefit of CDK4/6 inhibition plus radiation therapy in newly diagnosed, O^6^-methylguanine DNA methyltransferase (MGMT)-unmethylated glioblastoma. In fact, CDK4/6 inhibitor monotherapy has not demonstrated efficacy in solid tumors. We aimed at discovering response modulators to CDK4/6 inhibition, paving the way for rational combination therapies.

**Methods:**

We conducted genome-wide CRISPR-Cas9 screens in human glioma cell lines and stem-like cells (LN229, LN18, LNZ308, T98G, and GS-9) under CDK4/6 inhibition, employing knockout (Brunello library) and activation strategies (Calabrese library), followed by genetic and pharmacological validation of selected candidate genes *in vitro* and *ex vivo* (primary cultures) as well as the investigation of 1 functionally instructed combination therapy *in vivo*.

**Results:**

Loss of *AMBRA1* and gain of function of *CCNE1* reduced sensitivity to CDK4/6 inhibition in glioma cells, whereas disruption of checkpoint kinase 1 (*CHEK1*) or *FAM122A* resulted in synthetic lethality in combination with CDK4/6 inhibition. *AMBRA1*-deficient glioma cells exhibited increased sensitivity to CHK1 inhibition, revealing a context-specific vulnerability. Combined inhibition of CHK1 and CDK4/6 led to synergistic antiglioma activity in vitro, ex vivo, and in vivo.

**Conclusions:**

Our data identify *AMBRA1*, *CCNE1*, *CHEK1*, and *FAM122A* as potential molecular modifiers of CDK4/6 inhibition response in experimental glioma and provide a biological rationale for combinatorial targeting with CDK4/6 inhibition in glioblastoma.

Key Points

- *AMBRA1* loss or *CCNE1* activation reduce the sensitivity to CDK4/6 inhibition.
- Disruption of checkpoint kinase 1 (*CHEK1*) or *FAM122A* induces synthetic lethality in combination with CDK4/6 inhibition.
- Combined inhibition of CHK1 and CDK4/6 displays synergistic anti-glioma activity.

Importance of the Study

This study applied a genome-wide CRISPR-Cas9-based functional genomics approach to discover modulators of the therapeutic efficacy of the cyclin-dependent kinase 4/6 (CDK4/6) inhibitor abemaciclib in experimental glioma. Using genetic, pharmacological, and functional experimental approaches for target validation *in vitro*, *ex vivo*, and *in vivo*, we identified cell cycle and DNA damage response-associated molecular modifiers of response under CDK4/6 inhibition. The combination of CDK4/6 and checkpoint kinase 1 inhibition represents a therapeutic strategy from a translational perspective, particularly in activating molecule in the context of autophagy and Beclin-1 regulator 1 (*AMBRA1*) loss.

Glioblastoma (GB) is an aggressive primary tumor of the central nervous system,^1^ with a median survival in the range of 1.5 years despite multimodal treatment strategies involving maximal surgical resection, alkylating chemotherapy, and tumor treating fields.^2–5^ A hallmark molecular alteration in GB is dysregulation of the retinoblastoma (*RB1*) pathway,^6^ which governs the G1/S transition of the cell cycle through key components including cyclin-dependent kinase 4/6 (CDK4/6), CDKN2A/B, and *RB1* itself.

Nearly 80% of primary GBs harbor disruptions such as *CDKN2A/B* homozygous deletion or hypermethylation, *CDK4/6* amplification or overexpression, or *RB1* mutation/loss.^6^^,^^7^ While loss of *RB1* renders tumors resistant to CDK4/6 inhibition, mutations and/or amplifications in cell cycle inhibitors or kinases driving cell cycle progression within the G1 phase suggest a preferential dependency of GB.^8^ CDK4/6 inhibitors such as palbociclib, ribociclib, and abemaciclib have been evaluated in GB.^9–11^ Among these, abemaciclib exhibits broader kinase inhibition and superior blood-brain barrier penetration.^12^^,^^13^

On a molecular level, CDK4/6 inhibition effectively ­suppresses RB phosphorylation, induces G1 arrest, and attenuates tumor growth in patient-derived xenografts, particularly within the proneural subtype of GB.^12^^,^^14^ Acquired resistance to CDK4/6 inhibition limits the efficacy^15^^,^^16^ and is often mediated by reactivation of cell-cycle regulators such as CCNE1 or by compensatory oncogenic signaling via PI3K/mTOR, which mitigates the cytotoxic effects of CDK4/6 inhibition.^17^^,^^18^ Clinical trials have thus far shown limited therapeutic benefit in glioblastoma. A phase II clinical trial of palbociclib in RB1-intact GB failed to improve survival.^11^ Furthermore, the NOA-20 trial did not report a progression-free survival benefit for palbociclib plus radiotherapy in molecularly selected patients.^19^ The limited efficacy of CDK4/6 inhibitors as systemic monotherapy, observed not only in glioblastoma but also across most solid tumors, highlights the need for rationally designed combination strategies. Such approaches require a robust molecular and biological rationale to ensure translational relevance. To address this knowledge gap, we employed genome-wide CRISPR-Cas9-based functional genomics screens to identify novel synthetic lethal interactions and key resistance mechanisms. This strategy revealed actionable vulnerabilities that may guide combinatorial therapies aimed at overcoming resistance and improving the clinical efficacy of CDK4/6 inhibition.

## Methods

### CRISPR-Cas9 Knockout and Activation Screens

We employed CRISPR-Cas9 screens to investigate gene alterations that either enhance or suppress the activity of abemaciclib in glioma. We utilized a genome-wide CRISPR-Cas9 knockout library (Brunello, comprising 76 441 sgRNAs targeting 19 114 genes) and a CRISPR-dCas9-VP64 activation library (Calabrese P65-HSF, comprising 56 762 sgRNAs targeting 18 885 gene promoters).^20^^,^^21^ Glioma cells expressing Cas9 or dCas9-VP64 were transduced with lentiviral libraries, ensuring 500× coverage at a transduction efficiency of 30%. Transductions were performed in technical duplicates. After 24 h, puromycin was applied at a predetermined concentration for 5 days to select for transduced cells, with transduction efficacy monitored via an in-line assay. On day 7, cells equivalent to a 500× library coverage were split into drug and dimethyl sulfoxide (DMSO) control groups. Drug treatments involved abemaciclib at predetermined concentrations to induce complete cytotoxic (1.5-6 µM) (activation screens) or cytostatic (100 nM) (knockout screens) effects, aiming to identify genes associated with resistance or synthetic lethality, respectively. Throughout the 14-day treatment period, cells were passaged under continuous drug exposure. In control groups treated with DMSO, a minimum of 500× library coverage was preserved at all stages. After 2 weeks, genomic DNA was extracted using Qiagen’s QIAamp Blood Maxi, Midi, or Mini kits, depending on cell quantity, and subjected to next-generation sequencing for analysis.^20^

### CRISPR-Cas9 Screen Analysis

Sequencing data from the plasmid pools corresponding to the Brunello and Calabrese sgRNA libraries, used to generate the lentiviral particles in this study, were obtained from the Broad Institute. Following next-generation sequencing, quality control was performed using FastQC (v0.11.9). Reads were aligned to the respective sgRNA reference libraries and quantified using PoolQ (v3.4.3). Subsequent screen analysis was performed using the nf-core/crisprseq workflow (v2.0.0; https://nf-co.re/crisprseq/2.0.0). As part of this pipeline, log2 fold changes in sgRNA abundance between treatment (abemaciclib or DMSO) and plasmid reference samples were calculated. To correct for gene-independent effects, normalized read counts were adjusted using crisprcleanR (v3.0.0). The resulting data were analyzed using the MAGeCK MLE algorithm implemented in MAGeCK (v0.5.9), which applies maximum-likelihood estimation to infer gene-level effects and reports beta scores alongside false discovery rate (FDR) statistics for each gene.^22^

For knockout screens, no additional normalization was applied, as crisprcleanR incorporates genewise median ratio normalization. Comparative analyses were guided by condition-specific design matrices, contrasting either treatment conditions against DMSO controls or plasmid references. To evaluate broader trends across all profiled cell lines, additional aggregated analyses were performed, comparing all abemaciclib-treated samples either to plasmid controls or to pooled DMSO-treated counterparts. These comparisons provided a consolidated view of screen-wide effects under drug treatment. Data visualization and downstream interpretation were conducted using MAGeCKFlute (v3.16). The investigated genes scored an FDR<0.1 and a beta-value<0.5. We further excluded essential genes for the knockout screens.^23^

### RNA Sequencing

Detailed RNA sample preparation, sequencing, and processing were performed (Supplementary Material). In brief, glioma cell lines were treated with abemaciclib or DMSO controls, and the total RNA was isolated for mRNA enrichment and library preparation. Postsequencing, Nextflow-based data processing was implemented. Differential gene expression analysis was conducted with DESeq2 using normalization and filtering criteria, with models accounting for treatment, cell line effects, and interaction terms.^24^ Clustering of candidate genes was then performed to reveal expression patterns.

### Genetic Validation of CRISPR Screen Hits

Cas9 or dCas9-VP64 derivatives were generated by transducing with the lentiviral vector lentiCas9-Blast (Addgene #52962) or lentidCas9-VP64 (Addgene #61425) and selection by blasticidin. Identified gene hits from CRISPR-Cas9 activation or knockout screens were validated by cloning selected sgRNAs from the Calabrese or Brunello libraries into pXPR_502 (gift from John Doench & David Root, RRID: Addgene_96923) or lentiGuide-Puro (gift from Feng Zhang, RRID: Addgene_52963) vectors, respectively, using Golden Gate Assembly Kit (BsmBI-v2) (New England Biolabs, Inc.). Lentiviral particles were produced using HEK293FT cells in combination with psPAX2 (Addgene #12260) and pMD2.G (Addgene #12259) plasmids. Glioma cell lines expressing dCas9-VP64 (activation) or Cas9 (knockout) were subsequently transduced with viral supernatants using spinfection. Forty-eight hours after transduction, transduced cells were selected using puromycin for 5 days.

### Acute Cytotox and Clonogenic Survival Assays

Assays were performed as previously described^25^ and are detailed in Supplementary Material S1.

### Long-Term Dose Response/Proliferation Assays

For the 12-day drug cell proliferation assays, we seeded the cells at their optimal cell density (1×10^5^ cells for LN229, LN18; 2×10^5^ cells for LNZ308, T98G and 5×10^5^ cells for GS-2, GS-9) per well in 6-well plates or 3×10^5^ cells (2.3×10^5^ cells for LN229, LN18; 4×10^5^ cells for LNZ308, T98G and 1×10^6^ cells for GS-2, GS-9) per flask in T25 flasks in triplicates and treated with corresponding monotherapies or drug combinations. Cells were treated once with the indicated drug, and the drug was only replaced at the time of reseeding after every 4 days. Cell numbers were determined on day 4, day 8, and day 12 postseeding. We used DMSO-treated cells as a control.

### Synergy Assays

Cytotoxic synergism was assessed between the 2 drugs of interest in a 96-well plate setting as has been described previously.^25^ Further details are outlined in Supplementary Material S1.

### Cell Cycle Analysis by Flow Cytometry

Cells were treated with either abemaciclib or DMSO, fixed, and stained with propidium iodide. DNA content was analyzed, and data were processed with FlowJo software. Further details are outlined in Supplementary Material S1.

### BrdU Incorporation Assays

Cells were pulsed with BrdU and subsequently fixed in ethanol. DNA denaturation and neutralization were followed by anti-BrdU staining and counterstaining with propidium iodide. Data were processed with FlowJo software (Supplementary Material S1).

### Apoptosis Analysis

We performed apoptosis analysis using the BD Annexin V/PI detection Kit according to the manufacturer’s protocol (Supplementary Material S1).

### CDK2-Cyclin E1 Kinase Activity Assays

We used a custom CDK2-Cyclin E1 lysate activity kit from AssayQuant, according to the manufacturer’s protocol (Supplementary Material S1).

### Co-Immunoprecipitation and Alpha Fold Prediction

We performed Co-immunoprecipitations followed by SDS-PAGE using FLAG and FLAG-AMBRA1 expressing glioma cells. ColabFold (v1.5.2), implementing the AlphaFold2 algorithm, was used to model protein complexes and predict interactions. Further details are outlined in Supplementary Material S1.

### Immunoblot

Protein lysates were extracted, denatured, and resolved by SDS-PAGE. Proteins were transferred to nitrocellulose membranes, blocked, and probed with primary antibodies overnight. Horseradish peroxidase (HRP)-conjugated secondary antibodies and chemiluminescent detection were used for visualization using the ChemiDoc system (Supplementary Material S1).

### Glioma Cell Lines and Glioma Stem-Like Cultures

Long-term glioma cell lines (LN229, LN18, LNZ308, and T98G) and stem-like cell lines (GS-2, and GS-9) were cultured as described previously^25^ (Supplementary Material S1).

### Primary Glioblastoma Cultures

Primary cultures were generated^25^^,^^26^ from human glioblastoma tumors and treated as indicated, with cytotoxicity measured via CellTiter-Blue. For detailed procedure, see Supplementary Material S1.

### Patient-Derived Microtumor Models

Patient-derived microtumors were generated from freshly resected human glioblastoma as described^27^ (Supplementary Material S1) and treated with indicated drugs or combinations.

### Orthotopic Murine Glioma Models

Injection of human glioma cells (75 × 10^3^ LN229) into the right striatum of CD1nu/nu mice was performed as described^25^^,^^26^ (Supplementary Material S1).

### Statistical Analysis

All statistical analyses were performed in GraphPad Prism 9 or R (v4.0.5). To compare 3 or more groups, we used 2-way ANOVA with Tukey’s test for multiple comparisons. Differences are considered significant at *P *< .05. In all graphs, data are presented as mean ± SD, and details of normalization are provided in figure legends. A biostatistical assessment was performed for the animal experiments. Sample size calculations were based on an expected power of 80%, assuming a normal distribution and SD derived from prior experiments measuring survival as the time to onset of neurological symptoms. Survival analyses were conducted using the log-rank test.

## Results

### CRISPR-Cas9 Screens Reveal Modulators of Response to CDK4/6 Inhibition in Human Glioma Models

We performed genome-wide CRISPR-Cas9 knockout (Brunello library) and activation (Calabrese library) screens in human glioma cell lines (LN229, LN18, T98G, and LNZ308) and neurospheres (GS-9) under abemaciclib treatment (model overview: Figure S1A). These models represent both long-term glioma and glioma stem-like cell lines that show differential sensitivities toward CDK4/6 inhibition, potentially due to differing cytogenetic and transcriptional events within the Cyclin D-CDK4/6-RB1 axis (Figures S1B and C and S2A-E). To capture resistance (activation) and synthetic lethal (knockout) interactions, abemaciclib concentrations were optimized for each cell line to induce either cytostatic or cytotoxic responses, respectively. CRISPR screens were analyzed using MAGeCK MLE to identify significant hits within individual cell lines as well as those consistently scored across diverse genetic backgrounds (model overview: Figure S4A).

**Figure 1.**
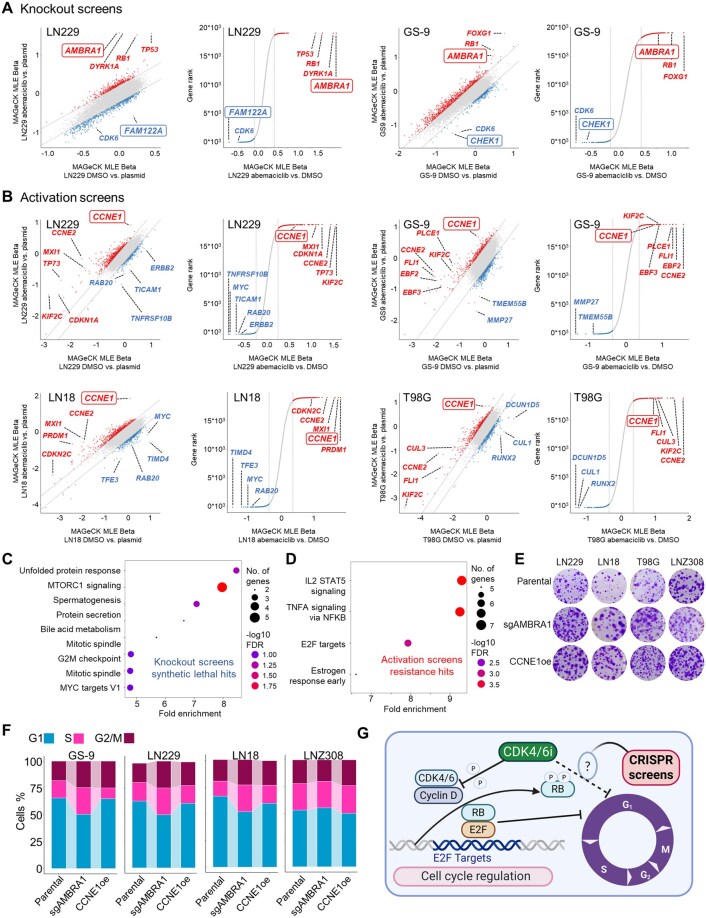
CRISPR-Cas9 screens reveal genetic modifiers of CDK4/6 inhibition response in human glioma models. (A) Scatter and rank plots of MAGeCK MLE results comparing Brunello sgRNA distributions from DMSO or abemaciclib-treated LN229 (left) (100 nM abemaciclib) and GS9 cells (right) (100 nM abemaciclib) to the plasmid library pool. (B) Scatter and rank plots of MAGeCK MLE results comparing Calabrese sgRNA distributions from DMSO or abemaciclib-treated LN229 (left, top row) (3 μM abemaciclib), GS-9 (right, top row) (6 μM abemaciclib), LN18 (left, bottom row) (2 μM abemaciclib), and T98G (right, bottom row) (1.5 μM abemaciclib), cells to the plasmid library pool. (C) Hallmark gene set enrichment analysis of drug-specific, uniformly scoring, negatively selected hits from the knockout screens, that is, synthetic lethal candidate genes. (D) Hallmark gene set enrichment analysis of drug-specific, uniformly scoring, positively selected hits from the activation screens, That is, resistance candidate genes. (E) Clonogenicity of adherent glioma cell lines. (F) Alluvial plots illustrating changes in cell cycle distribution of glioma cells upon loss of *AMBRA1* or gain of *CCNE1*. (G) Graphical summary of the proposed approach focusing on CDK4/6 cell-cycle machinery. Created in BioRender (https://BioRender.com/jbcv017). Statistics are derived from maximum likelihood estimation (A, B) and a hypergeometric test (C, D).

For knockout screens, we confirmed strong depletion of known common essential genes in DMSO-treated controls, indicating good screening performance (Figure S5). We selected significantly depleted (negatively selected) and enriched (positively selected) genes under abemaciclib treatment as compared to the DMSO control at an FDR<10%. Notably, the ubiquitin ligase receptor, activating molecule in BECN1-regulated autophagy protein 1 (*AMBRA1),* emerged as the top positively selected gene in our knockout screens (Figure 1A), indicating its loss may confer resistance to CDK4/6 inhibition. Other positively selected hits included retinoblastoma 1 (*RB1)*, dual specificity tyrosine phosphorylation-regulated kinase 1A (*DYRK1A)*, tumor protein 53 (*TP53)*, and forkhead box G1 (*FOXG1)*, which are implicated in cell cycle regulation and survival pathways. Among the negatively selected genes, cyclin-dependent kinase 6 (*CDK6)*, family with sequence similarity 122, member A (*FAM122A)*, and checkpoint kinase 1 (*CHEK1)* exhibited the strongest depletion under treatment, suggesting synthetic lethality with abemaciclib. Activation screens performed in parallel identified cyclin E1 (*CCNE1*) as the top resistance-promoting gene (Figure 1B and Figure S4B). Additional hits included cyclin E2 (*CCNE2)*, MAX interaction 1 (*MXI1)*, kinase family member 2C (*KIF2C)*, and PR domain containing 1 (*PRDM1)*. By focusing on genes with consistent scoring patterns, we identified candidates that may influence the cellular response to abemaciclib across different genetic backgrounds of the cell lines. Pathway enrichment analysis of these screen hits (Figure 1C and D) highlighted consistent depletion of MYC targets, MTORC1 signaling, G2M checkpoint, and mitotic spindle signatures in negatively selected knockout hits, whereas enriched hits in activation screens were associated with IL2 STAT5 signaling, TNF alpha signaling via NFKB, and E2F targets activation.

### G1 Phase Cell Cycle Regulators AMBRA1 and CCNE1 Are Key Modulators of Response to CDK4/6 Inhibition

We next investigated the ability of selected genetic alterations to confer resistance to abemaciclib treatment, focusing on loss-of-function of *AMBRA1* and gain-of-function of *CCNE1* as guided by the CRISPR screening approach. First, we analyzed the effect of genetic alterations (Figure S6A and B) on cell cycle distributions of glioma cell models. Flow cytometry analysis revealed an increased S and G2M-phase population in *AMBRA1*-deficient (sgAMBRA1) and *CCNE1* overexpressing (CCNE1oe) cells across all lines, except in LNZ308 (Figure 1F), which was further supported by enhanced clonogenicity in 3 of the 4 adherent glioma lines, with minimal effect in LNZ308 (Figure 1E). Western blot analysis confirmed these cell cycle-associated changes, showing increased expression of D-type cyclins upon *AMBRA1* loss in LN229, LN18, and GS-9 cells but not as strongly in LNZ308 (Figures S6C and D and S7).

**Figure 2.**
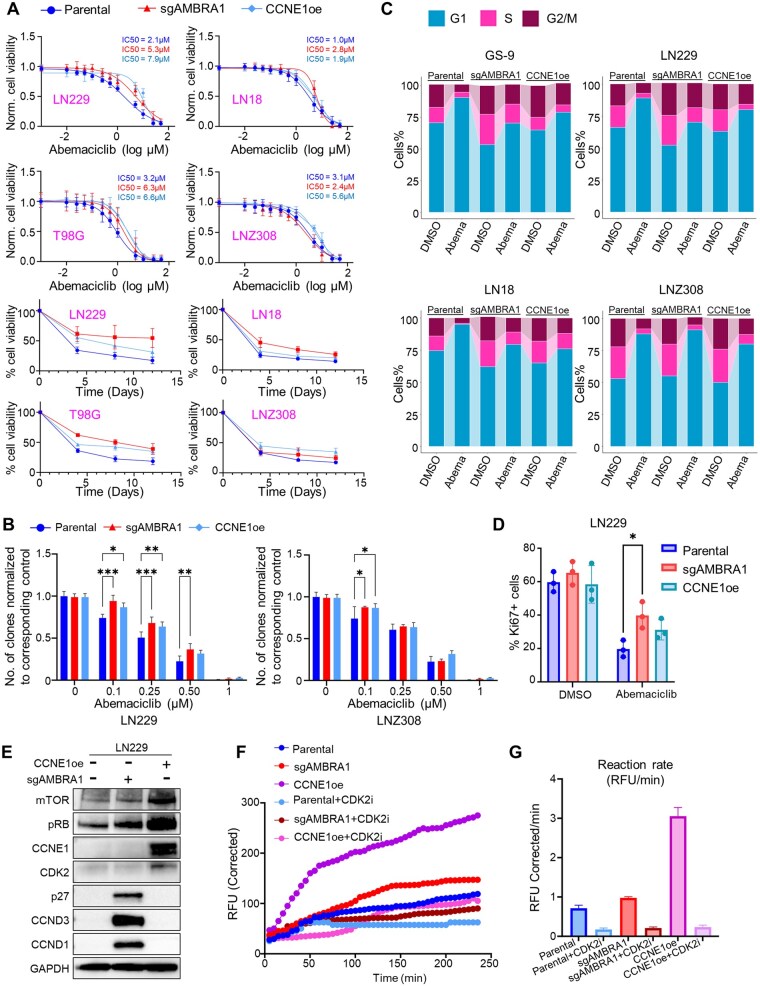
*AMBRA1* and *CCNE1* function as key modulators of CDK4/6 inhibitor sensitivity in glioma. (A) Acute (72 h) (top) and long-term (12 days) (bottom) dose response curves illustrating the viability of glioma cell lines in parental, *AMBRA1* knockout, or *CCNE1* overexpressing conditions under abemaciclib treatment. Values are shown as the viability normalized to/percent of control. (B) Analysis of clonogenic survival assays examining the response of parental, sgAMBRA1, and CCNE1oe LN229 and LNZ308 cells to increasing concentrations of abemaciclib. Quantification is shown as the number of colonies normalized to control. (C) Quantification of cell cycle distribution for distinct glioma cells under abemaciclib treatment. (D) Flow cytometric analysis of Ki67-positive, proliferative cells in parental, *sgAMBRA1*, or *CCNE1*-overexpressing LN229 cells after treatment with DMSO or abemaciclib. (E) Immunoblots comparing basal expression of associated proteins in LN229 parental, sgAMBRA1, and CCNE1oe cells (*n*=3). (F) CDK2/Cyclin E1 kinase activity curves from LN229 cell lysates. RFU indicates relative fluorescence unit measured over 240 min and corrected to the control. (G) Bar plot representing reaction rate, that is, the RFU measures per minute, comparing all 3 constructs under basal and CDK2i-treated conditions. Two-way ANOVA with Tukey’s post hoc comparisons; *P<.05, **P<.01, ***P<.001. Data represent mean ± SD from at least 3 independent experiments.

Next, we assessed the effect of *AMBRA1* and *CCNE1* genetic alterations on the sensitivity of glioma cells to abemaciclib treatment by employing both short-term (72 h) and long-term (12 days) dose-response assays. Both *AMBRA1* depletion and *CCNE1* overexpression lowered sensitivity to abemaciclib treatment in a context-dependent manner (Figure 2A). While both genetic conditions in LN229, LN18, GS-9, and T98G cells exhibited reduced sensitivity under CDK4/6 inhibition, LNZ308 cells displayed a comparatively smaller effect size, indicating that the magnitude of sensitivity varied among cell lines.

Clonogenic survival assays further supported this context-dependent effect. In LN229 cells, both *AMBRA1* depletion and *CCNE1* overexpression reduced sensitivity to abemaciclib, whereas LNZ308 cells again showed a less pronounced response (Figure 2B).

Next, we performed cell cycle analysis under abemaciclib and vehicle (DMSO) treatment. Apart from LNZ308, all *AMBRA1*-deficient cell models presented a significantly higher fraction of cycling cells (S phase and G2/M phase) (Figure 2C and Figure S8A and B). Both LN229 and GS-9 cells displayed a decreased apoptotic response (Figure S9) upon CDK4/6 inhibition as compared to the corresponding parental cells. Similar effects were seen for *CCNE1*-overexpressing cells, but on a smaller scale as compared to *AMBRA1*-deficient cells (Figure 2C and Figure S8A and B). Moreover, loss of *AMBRA1* or gain of function of *CCNE1* led to maintained Ki67 expression under CDK4/6 inhibition as compared to parental cells (Figure 2D).

E-type cyclins, together with CDK2, potentially facilitate S phase entry as an alternate mechanism to D-type cyclin-CDK4/6-dependent cell cycle progression,^28^^,^^29^ and we observed sustained CDK2 expression in *CCNE1* overexpressing cells under CDK4/6 inhibition compared to parental cells (Figure S11). Although both *CCNE1* overexpression and *AMBRA1* loss attenuated G1 arrest induced by CDK4/6 inhibition, we did not detect any interpaly between CDK2/Cyclin E1 activity in these 2 models (Figure 2E-G and Figure S12). Notably, only CCNE1oe cells displayed elevated CDK2 activity relative to parental and sgAMBRA1 cells, suggesting that these alterations drive cell cycle reentry through distinct mechanisms.

**Figure 3.**
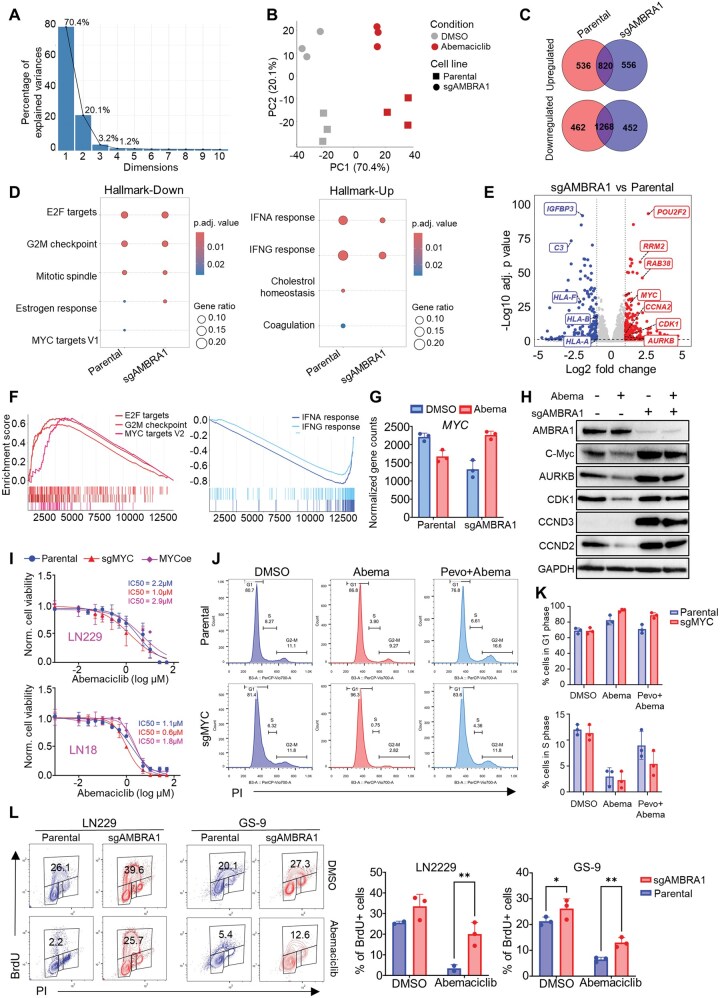
*AMBRA1* loss remodels gene expression upon CDK4/6 inhibition. (A) Scree plot illustrating the percentage of variance explained by the first 10 principal components within gene expression profiles from parental and sgAMBRA1 LN229 cells treated with DMSO or abemaciclib. (B) Scatter plot illustrating separation of parental and sgAMBRA1 LN229 cells upon CDK4/6 inhibition according to the first 2 principal components. (C) Venn diagrams showing overlap of differential gene expression signatures in LN229 cells upon CDK4/6 inhibition, either upregulated (log2FC > 0.58, *P*<.05) or downregulated (log2FC < −0.58, *P*<.05) in parental and sgAMBRA1 cells. (D) Overrepresentation analysis using hallmark signatures for genes upregulated or downregulated in abemaciclib vs DMSO-treated parental and *sgAmbra1* LN229 cells. (E) Volcano plot illustrating genes that are differentially regulated by CDK4/6 inhibition in parental and *sgAMBRA1* LN229 cells. (F) Gene set enrichment analysis using hallmark signatures for differentially affected genes in parental and sgAMBRA1 cells. (G) Normalized counts of *MYC* in LN229 cells under abemaciclib treatment. (H) Immunoblots displaying expression of c-Myc, AURKB, CDK1, CCND2, CCND3, AMBRA1, and GAPDH in parental and sgAMBRA1 cells under DMSO or abemaciclib in LN229 cells (*n*=3). (I) Dose response curves illustrating the viability of LN229 and LN18 cell lines in parental, *MYC* knockout, or *MYC* overexpressing conditions under 72 h abemaciclib treatment. (J) Flow cytometry-based cell cycle analysis of parental vs sgMYC LN229 cells either treated with DMSO, abemaciclib, or pevonedistat plus abemaciclib for 24 h. (K) Quantification of percentage of cells in G1 and S phase from (J). (L) Flow cytometry-based measurement of G1, S, and G2-M phase cell fractions in parental and sgAMBRA1 LN229 and GS-9 cells. Quantification illustrates changes in the fraction of BrdU-positive, S-phase cells. Two-way ANOVA with Tukey’s post hoc comparisons; *P<.05, **P<.01. Data represent mean ± SD from at least 3 independent experiments.

AMBRA1 has recently been described as the master regulator of D-type cyclin stability.^30^^,^^31^ To assess how AMBRA1 loss affects response to CDK4/6 inhibition, we compared gene expression profiles of parental and sgAMBRA1 LN229 cells treated with abemaciclib (Figure 3). In both models, the dominant transcriptional effect (70.4% of variance) reflected abemaciclib-induced G1 arrest and suppression of proliferative gene signatures, indicating a similar primary drug response regardless of AMBRA1 status (Figure 3A-D and Figure S13A and B). However, principal component analysis showed that the second component (20.1% variance) separated parental and AMBRA1-deficient cells, indicating differential transcriptional responses to treatment. Interaction term-based differential expression analysis revealed sustained expression of key cell cycle regulators, including *MYC, RRM2, CCNA2, CDK1*, and *AURKB* in sgAMBRA1 cells under abemaciclib treatment (Figure 3E). Consistently, gene set enrichment analysis showed persistent activation of pathways associated with G2/M checkpoint control, E2F targets, and cell cycle progression through MYC targets (Figure 3F and Figure S13C). Among these genes, *MYC* emerged as a representative marker of sustained proliferative signaling, with elevated transcript levels in sgAMBRA1 cells after abemaciclib treatment (Figure 3G). Correspondingly, c-Myc, AURKB, CDK1, Cyclin D2, and Cyclin D3 protein levels were increased in AMBRA1-deficient cells compared to parental controls (Figure 3H and Figure S14A).

To determine whether *MYC* drives the resistance phenotype associated with *AMBRA1* loss, we generated *MYC* knockout (sgMYC) and overexpressing (MYCoe) models (Figure S15A). Dose response assays demonstrated that knockout of *MYC* increased sensitivity to CDK4/6 inhibition, whereas overexpression led to reduced sensitivity (Figure 3I and Figure S15B). To mimic *AMBRA1* loss in MYC-deficient cells, we inhibited CRL4 activity using pevonedistat (Figure 3J and K and Figure S15C and D). In parental (MYC-proficient) cells, abemaciclib induced a G1 arrest that was reversed by pevonedistat, phenocopying the AMBRA1-loss effect. In contrast, sgMYC cells exhibited stronger G1 arrest under abemaciclib, indicating increased sensitivity, and the inhibition of CRL4 activity still reversed the effect of CDK4/6i. These findings suggest that MYC alone is not the primary driver of the resistance phenotype observed upon AMBRA1 loss. Furthermore, BrdU incorporation assays indicated sustained DNA synthesis in *AMBRA1*-deficient cells under CDK4/6 inhibition in both LN229 and GS-9 models, confirming continued cell cycle activity (Figure 3L). LNZ308, on the other hand, did not exhibit similar BrdU incorporation (Figure S14C and D).

Collectively, our results identify *AMBRA1* as a critical modulator of CDK4/6 inhibitor response in glioma cells, whose loss enables a partial bypass of CDK4/6-mediated cell-cycle arrest through maintenance of proliferative signaling, which may cooperate with *MYC* to sustain cell-cycle progression despite CDK4/6 inhibition.

### AMBRA1 Loss Sensitizes Glioma Cells to CHK1 Inhibition and Irradiation

**Figure 4.**
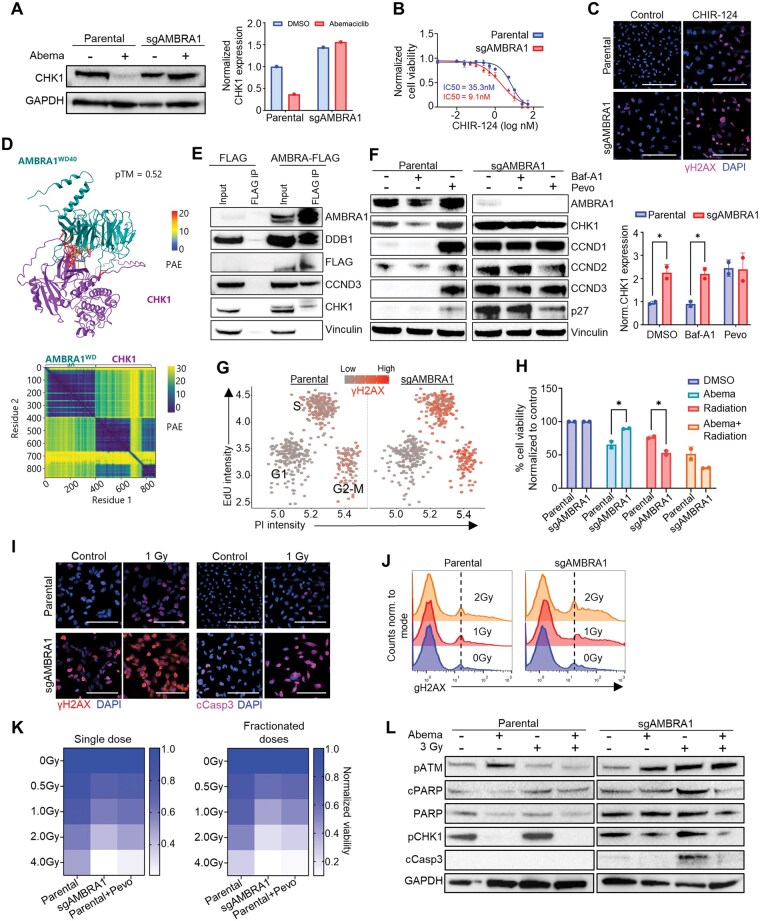
*AMBRA1* loss sensitizes glioma cells to CHK1 inhibition and ionizing radiation. (A) Immunoblot analysis of CHK1 protein levels in LN229 parental and sgAMBRA1 cells exposed to either DMSO control or abemaciclib for 48 h; GAPDH serves as a loading control; quantified on the right. (B) Cell viability of parental vs sgAMBRA1 cells after 72 h of CHIR-124 treatment. (C) Immunofluorescence staining of γH2AX in LN229 cells treated with CHIR-124 or the respective control, pink displays γH2AX, and blue displays DAPI. (D) Complex structure prediction of AMBRA1WD40 with CHK1 using AlphaFold2_mmseqs2. Top: cartoon representations of the predicted heteromeric protein complexes. Interchain AlphaFold2 contacts (<8 Å) are depicted as straight lines and colored according to the predicted alignment error (PAE). AMBRA1 residues involved in DDB1 binding are highlighted in green. Predicted template modeling scores (pTM) are shown. Bottom: pairwise PAE scores for each protein complex. (E) Co-immunoprecipitation analyses followed by western blotting to assess selected candidate AMBRA1 interactors and substrates. Previously reported AMBRA1 interactors and substrates (DDB1, cyclin D1, and cyclin D3) were included as controls (*n*=3). (F) Immunoblot analyses of cyclin D1/2/3, CHK1, and p27 in LN229 cells under parental and *AMBRA1* knockout conditions (*n*=3). Cells were treated with DMSO, 0.4 μM Baf-A1, or 1 μM MLN4924 for 12 h. Right: Quantification of CHK1 expression levels relative to the corresponding DMSO control. (G) Scatter plots representing γH2AX, EdU, and PI intensities in parental vs sgAMBRA1 LN229 cells. (H) Normalized cell viability of parental and AMBRA1-deficient LN229 cells under abemaciclib, radiation, and their combination treatment. (I) Immunofluorescence staining of γH2AX (red) and cleaved caspase 3 (pink) in LN229 cells treated with ionizing radiation. (J) FC measured γH2AX intensity in cells treated with increasing dosages of irradiation. (K) Heatmap illustrating cell viability of LN229 cells under single or fractionated dosages of irradiation. (L) Immunoblot analysis of parental and *AMBRA1* knockout LN229 cells treated with single agents or combined abemaciclib and radiation treatment, showing expression of apoptosis and DNA damage response-associated markers (*n*=3). Scale bars 50 µm, at 40× magnification. Two-way ANOVA with Tukey’s post hoc comparisons; *P<.05. Data represent mean ± SD from at least 3 independent experiments.

We next exploited therapeutic strategies that could overcome resistance to CDK4/6 inhibition associated with *AMBRA1* loss in glioma cell lines. It has been shown previously that loss of AMBRA1 leads to replication stress and sensitivity to CHK1 inhibition.^31^ Of note, loss of AMBRA1 in LN229 cells induces increased levels of CHK1, and these levels are maintained upon CDK4/6 inhibition (Figure 4A and Figure S17A).

Furthermore, sgAMBRA1 cells showed increased sensitivity to the CHK1 inhibitor CHIR-124, as demonstrated by a pronounced reduction in cell viability compared to parental cells in acute viability assays (Figure 4B). CHK1 inhibition in the context of AMBRA1 loss also resulted in significantly increased DNA damage, as shown by elevated γH2AX immunofluorescence staining (a marker of double-strand breaks; Figure 4C), indicating enhanced replication stress under *AMBRA1* loss, consistent with previous reports (reference). Synergy analysis using CHIR-124 in combination with the CDK4/6 inhibitor abemaciclib revealed a stronger cytotoxic interaction in sgAMBRA1 cells than in parental counterparts (Figure S16A-D), suggesting that AMBRA1 loss enhances vulnerability to replication checkpoint disruption in combination with G1/S cell-cycle inhibition. The observed sensitivity to CHK1i was not a direct consequence of CDK4/6i, as indicated by no substantial changes in AMBRA1 levels upon CDK4/6i (Figure S10). Since AMBRA1 functions as a master regulator of Cullin-RING E3 ligase 4 (CRL4) complexes,^32^ we investigated whether AMBRA1 might directly interact with CHK1. Structural predictions using AlphaFold indicated a moderate interaction interface between AMBRA1 and CHK1 (Figure 4D and Figure S17B and C), which was further supported by co-immunoprecipitation experiments demonstrating physical association between the two proteins (Figure 4E). To dissect whether this interaction depended on AMBRA1’s role in autophagy or its CRL4 regulatory function, cells were treated with the autophagy inhibitor bafilomycin A1 or the CRL4 activity inhibitor pevonedistat. Notably, only pevonedistat treatment phenocopied *AMBRA1* knockout, resulting in elevated CHK1 and Cyclin D3 expression (Figure 4F). These findings indicate that AMBRA1 regulates CHK1 through its role in the CRL4 machinery rather than through autophagy.

Given that AMBRA1-deficient cells display increased replication stress and CHK1 dependency (Figure 4B, C, and G), and since radiation therapy induces DNA double-strand breaks and relies on intact checkpoint signaling for repair,^33^ we hypothesized that they may also exhibit heightened sensitivity to DNA damage induced by ionizing radiation. Indeed, cell viability assays confirmed that sgAMBRA1 cells were more susceptible to radiation compared to parental cells, and this effect was significantly enhanced when combined with abemaciclib (Figure 4H). We further observed increased γH2AX and cleaved caspase 3 levels in sgAMBRA1 cells even after low-dose radiation exposure (Figure 4I), consistent with replication stress induced enhanced cell death. Clonogenic survival assays supported these observations by showing pronounced dose-dependent reductions in clonogenicity in sgAMBRA1 cells exposed to 1 and 2 Gy of radiation, while parental cells retained higher survival (Figure S18A). Moreover, AMBRA1-deficient cells displayed increased γH2AX accumulation in S and G2/M populations following irradiation (Figure 4J and Figure S18D), consistent with unresolved replication-associated DNA damage. Importantly, pharmacological inhibition of CRL4 activity with pevonedistat in parental cells recapitulated the radiosensitization phenotype observed in sgAMBRA1 cells under both single-dose and fractionated radiation regimens (Figure 4K and Figure S18B and C). On the protein level, AMBRA1-deficient cells showed increased levels of cleaved caspase-3, cleaved PARP, and phosphorylated ATM (pATM), indicative of enhanced DNA damage signaling and apoptosis (Figure 4L and Figure S19A and B). Notably, phosphorylated CHK1 (pCHK1) levels were substantially elevated in sgAMBRA1 cells under radiation compared to parental cells. These results suggest that in the absence of AMBRA1, glioma cells experience heightened replication stress and accumulate DNA damage, particularly upon radiation or CHK1 pathway inhibition, leading to apoptotic cell death.

### Genetic Depletion of FAM122A or CHEK1 Sensitizes Glioma Cells to CDK4/6 Inhibition

**Figure 5.**
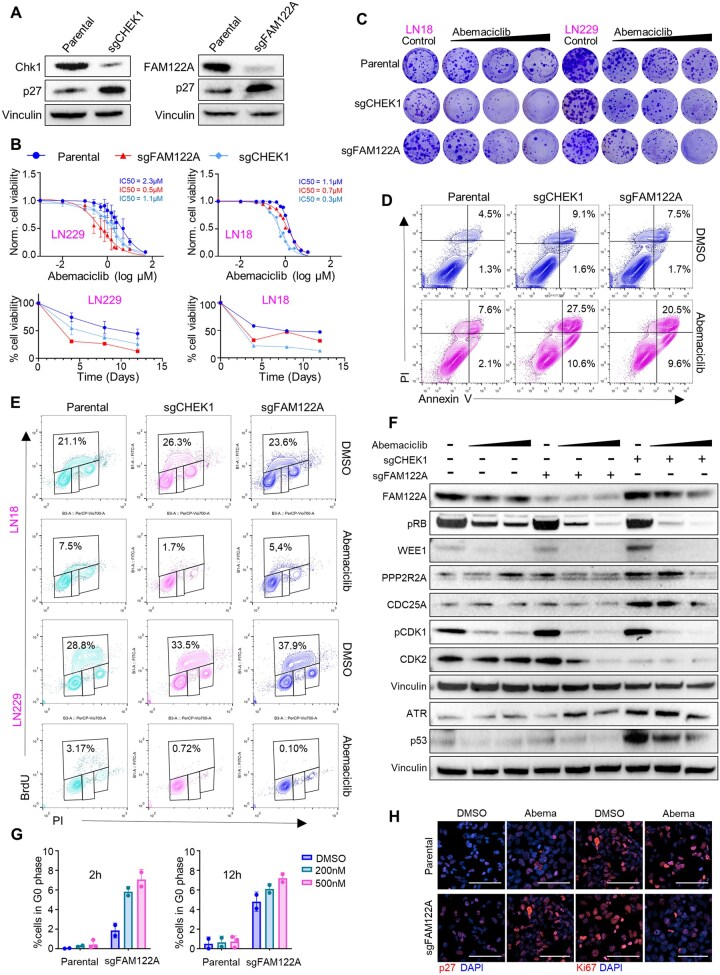
Genetic depletion of *FAM122A* or *CHEK1* sensitizes glioma cells to CDK4/6 inhibition. (A) Immunoblot analysis of CHEK1 and FAM122A knockout validation, also displaying p27 expression, and vinculin as a housekeeping control. (B) Clonogenic survival assays in parental, sgFAM122A, and sgCHEK1 cells under DMSO control or increasing dosages of abemaciclib. (C) Acute (72 h) (top) and long-term (12 days) (bottom) dose response curves showing cell viability of LN229 and LN18 parental, sgFAM122A, and sgCHEK1 cells treated with increasing concentrations of abemaciclib. Values are shown as viability normalized to the control. (D) Flow cytometric analysis of apoptosis using Annexin V/PI staining in LN18 cells with or without *FAM122A* or *CHEK1* deletion following DMSO or abemaciclib treatment for 48 h. (E) BrdU incorporation in LN229 and LN18 cells with or without *FAM122A* or *CHEK1* knockout, treated with DMSO or abemaciclib for 48 h. (F) Immunoblot assays on parental vs sgFAM122A vs sgCHEK1 cells either treated with DMSO or increasing dosages of abemaciclib; vinculin serves as the loading control (*n* = 3). (G) Bar graphs showing fraction of G0 cells treated with indicated abemacilcib concentrations. Cells were serum-starved for 2 or 12 h before adding serum-containing media and respective treatment. (H) Immunofluorescence staining of p27 (red) (left) and Ki67 (red) (right) in LN229 cells treated with DMSO or abemaciclib post serum-starvation. Scale bars 50 µm, at 40× magnification. Data represent mean ± SD from at least 3 independent experiments.

Next, we focused on synthetic lethal interactors of CDK4/6 inhibition, *FAM122A* and *CHEK1*, as identified in the knockout screening approach (Figure 1A). To functionally evaluate whether the loss of *FAM122A* or *CHEK1* enhances the sensitivity of glioma cells to CDK4/6 inhibition, we genetically depleted each gene using sgRNA-mediated knockout (Figure 5A and Figure S20A and B) and assessed the impact on cell cycle distribution and sensitivity toward CDK4/6 inhibition in glioma cell lines. Overall, we did not detect major changes in cell cycle distributions and associated proliferative capacity upon loss of either *FAM122A* or *CHEK1* (Figure S20C and D), which is in line with our screening results. In contrast, both *FAM122A* and *CHEK1*-deficient cells exhibited reduced cell viability in response to increasing concentrations of abemaciclib as compared to parental controls (Figure 5B and Figure S20E and F). These findings were further supported by clonogenic survival assays demonstrating enhanced sensitivity to CDK4/6 inhibition upon *CHEK1* or *FAM122A* loss in LN229 and LN18 cell lines (Figure 5C). Furthermore, apoptotic activity significantly increased under CDK4/6 inhibition in *CHEK1* and *FAM122A*-deficient glioma cells (Figure 5D and Figure S21A). In addition, G1 arrest upon CDK4/6 inhibition was markedly increased in cells lacking *CHEK1* or *FAM122A* (Figure 5E and Figure S21B-D).

Given the reported involvement of CHEK1 and FAM122A in the ATR-WEE1-PP2A signaling network,^34^^,^^35^ we examined pathway regulators following abemaciclib treatment (Figure 5F and Figure S23A and B). Both CHEK1 and FAM122A-deficient cells showed reduced RB1 phosphorylation compared to parental cells. CHEK1-deficient cells additionally displayed activation of p53 and ATR signaling, indicating a strong DNA damage response. While CDK2 levels remained unchanged in parental cells, they were reduced in both knockout models, accompanied by increased inhibitory phosphorylation of CDK1.

Notably, the PP2A regulatory subunit PPP2R2A decreased in knockout cells but increased in parental cells upon abemaciclib treatment. Consistent with a role for PP2A signaling, pharmacological PP2A inhibition with LB100 reduced abemaciclib sensitivity in FAM122A knockout cells, whereas PPP2R2A knockout cells showed increased sensitivity (Figure S24C and D). FAM122A-deficient cells also displayed a higher fraction of G0 cells (Figure 5G and Figure S25) and increased p27 and γH2AX following abemaciclib treatment (Figure 5H).

We next tested combination treatments targeting the ATR-CHK1-WEE1 pathway that further enhanced the response to CDK4/6 inhibition. Specifically, the WEE1 inhibitor adavosertib synergized with the CHK1 inhibitor CHIR-124, and abemaciclib combined with the PP2A inhibitor LB100 also showed synergy (Figure S24F). Analysis of the Cancer Genome Atlas glioma dataset further revealed that high FAM122A expression correlates with poorer overall survival and positively correlates with CHEK1 expression (Figure S26A and B).

Together, these findings suggest that disruption of the CHEK1-FAM122A axis perturbs ATR-CHK1-WEE1-PPP2R2A signaling, activates DDR-response activity, and increases glioma cell sensitivity to CDK4/6 inhibition.

### Combined Pharmacological Inhibition of CHK1 and CDK4/6 Exhibits Synergistic Antiglioma Efficacy In Vitro, Ex Vivo, and In Vivo

**Figure 6.**
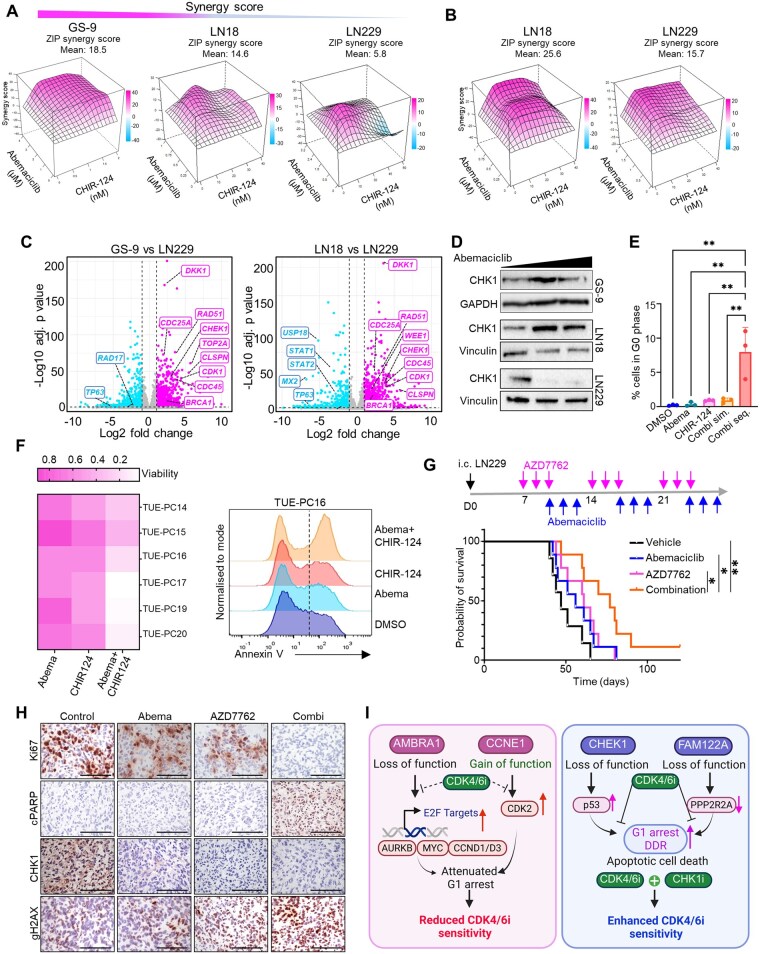
Combination of CDK4/6 and CHK1 inhibition synergistically reduces glioma cell viability. (A) Three-dimensional ZIP synergy plots showing the degree of synergy between abemaciclib and CHIR-124 in GS-9, LN18, and LN229 cell lines, both simultaneous (A) and sequential (B, CHIR-124 followed by abemaciclib) treatments. (C) Volcano plot of differentially expressed genes in GS-9 vs LN229 (left) and LN18 vs LN229 (right) cells under abemaciclib treatment; positive log_2_ fold change values correspond to genes that are relatively more expressed (either upregulated or less downregulated) in LN18 cells compared to LN229 cells. (D) Immunoblot analysis of CHK1 protein levels following treatment with abemaciclib for 48 h, vinculin, or GAPDH serves as a loading control. (E) FC measured fraction of LN18 cells in G0 phase treated with abemaciclib, CHIR-124, their simultaneous combination (Combi sim.), or sequential combination (Combi seq.). (F) Heatmap representing normalized viability scores across multiple patient-derived primary glioblastoma cell cultures treated with abemaciclib, CHIR-124, and their combination (left); apoptosis analysis of primary TUE-PC16 cells post 48 h of treatments with single agents, their combination, or DMSO control. (G) In vivo treatment scheme (top) and survival analysis (Kaplan-Meier, bottom) of CD1-nude mice bearing LN229 xenografts treated with abemaciclib, AZD7762 (CHK1 inhibitor), their combination, or the vehicle control (*n* = 9/treatment group). (H) Immunohistochemical analysis of murine brain tumors harvested from treated mice showing cells positively stained for Ki67, cleaved PARP, CHK1, and gH2AX. (I) Graphical abstract: Schematic summary of the proposed modulation of response to CDK4/6 inhibition in glioma. Created in BioRender (https://BioRender.com/k16etjz). Scale bars 100 µm, at 40× magnification. Statistical analysis for survival curves was performed with the log-rank test; comparisons indicate the combination vs all groups individually. *P_adjust_ < .05, **P_adjust_ < .01.

Given the availability of CHK1 inhibitors, we tested CHIR-124 and AZD7762 across multiple glioma cell lines (Figure S27A and B). All lines showed dose-dependent responses, although GS-2 and GS-9 were less sensitive to CHIR-124. We next evaluated combined CHK1 and CDK4/6 inhibition. Using the ZIP model, simultaneous treatment with abemaciclib and CHIR-124 showed synergistic growth inhibition in GS-9, LN18, and T98G cells, while LN229 and GS-2 exhibited only additive effects (Figure 6A and Figure S28A). Sequential treatment (CHK1 inhibition followed by abemaciclib) substantially increased synergy across all models (Figure 6B), including LN229. Clonogenic assays confirmed that the combination suppressed colony formation, particularly in LN18 cells (Figure S28B).

Transcriptomic profiling revealed that GS-9 and LN18 cells maintained elevated expression of cell cycle and DNA damage response genes, including *CHEK1*, *RAD51*, *WEE1*, *CDC25A*, *BRCA1*, and *CDK1*, under abemaciclib treatment compared to LN229 cells (Figure 6C, Figures S28C-G and S29). Consistently, CHK1 protein levels remained stable in GS-9 and LN18 but were strongly reduced in LN229 cells (Figure 6D), potentially explaining their differential sensitivity to CHK1 inhibition. Consistently, sequential treatment resulted in stronger G0/G1 cell-cycle arrest (Figure 6E and Figure S31A) and increased apoptotic cell death compared to simultaneous treatment (Figure S31B). Together, these findings align with prior reports indicating that CDK4/6 inhibition can induce replication stress, thereby increasing reliance on CHK1-mediated checkpoint activation for DNA repair and survival.^36^

In patient-derived glioma cultures (TUE-PC series), the combination reduced viability more effectively than monotherapies and increased apoptosis (Figure 6F and Figure S32A-D). Similar effects were observed in a patient-derived microtumor model (Figure S32F). Publicly available human single-cell RNAseq data^37^^,^^38^ further revealed distinct expression patterns of genes related to the CDK4/6 pathway (Figures S33-S36).

Next, we evaluated the in vivo efficacy of this combination in an orthotopic glioma xenograft model using LN229 cells. Mice bearing intracranial tumors were treated with vehicle, abemaciclib, the CHK1 inhibitor AZD7762, or the combination using a sequential treatment schedule (Figure 6G). Kaplan-Meier analysis showed significantly prolonged survival in the combination group compared to monotherapies or control (Figure 6G). Immunohistochemical analysis of tumor tissues revealed reduced proliferation (Ki67) and lower CHK1 levels across treatment groups, with the strongest effect in the combination group (Figure 6H). Notably, combination treatment also produced the highest levels of cleaved PARP and γH2AX, indicating increased DNA damage and apoptosis.

Together, these preclinical results demonstrate that co-targeting CDK4/6 and CHK1 exerts synergistic antiglioma effects in vivo and supports further clinical exploration of this combinatorial strategy.

## Discussion

CDK4/6 inhibitors have shown clinical activity in various solid tumors, mainly in combination therapies, and one major underlying feature of their molecular action is their capacity to induce a G1 phase cell cycle arrest.^36^^,^^39^ Yet, clinical experience of CDK4/6 inhibition in glioblastoma clinical trials has been disappointing so far,^11^^,^^19^ even in molecularly preselected patient populations.^19^ The limited efficacy of CDK4/6 inhibitors in brain tumors mainly reflects restricted CNS penetration and tumor-intrinsic resistance (e.g., RB loss, pathway redundancy), rather than suboptimal systemic dosing.^40^^,^^41^ Notably, the clinical doses used are within the therapeutic range, effective in HR+/HER2− breast cancer.^42^^,^^43^ We utilized abemaciclib, which demonstrates superior CNS penetration compared to palbociclib and ribociclib,^43^ although strategies to enhance its efficacy warrant further investigation.

Using genome-wide CRISPR-Cas9 knockout and activation screens, we identified *AMBRA1* and *CCNE1* as top mediators conferring resistance to CDK4/6 inhibition (Figure 1A and B). These findings are consistent with recent reports implicating both genes in regulating CDK4/6 inhibitor response across multiple cancer types.^44–47^ As a substrate receptor in the CUL4-DDB1 E3 ubiquitin ligase complex, AMBRA1 promotes cyclin D degradation, potentially restraining CDK4/6 activity.^30^^,^^32^ Accordingly, AMBRA1 loss resulted in enhanced D-type cyclin expression and persistent CDK4/6 signaling under both basal conditions as well as pharmacological inhibition of CDK4/6 (Figure 3H and Figure S6C and D). Functionally, AMBRA1 deficiency attenuated G1 arrest and maintained proliferative capacity under CDK4/6 inhibitor treatment (Figure 2A-D). The effects of AMBRA1 loss were observed across most cellular models but not in LNZ308 cells. This differential response likely reflects the distinct genomic context of this model, characterized by *CDK4* and *CCND1* amplification, elevated CDK4/Cyclin D1 activity, and low baseline AMBRA1 expression (Figures S1C and S2A-E). In such a setting, the CDK4/6 axis may already be maximally activated, limiting the additional impact of AMBRA1 loss. While abemaciclib induces partial cell cycle arrest, the absence of AMBRA1 allows a subset of proliferative programs to remain active, likely mediated through bypass activation of CDK1, AURKB, and downstream MYC signaling (Figure 3). This aligns with prior findings in melanoma, lung, and breast cancers, identifying the AMBRA1-Cyclin D-CDK4/6-RB axis as a pivotal resistance node.^48^^,^^49^

Contrasting our results for *AMBRA1* loss, *CCNE1* overexpression promoted resistance through activation of CDK2 signaling (Figure 2E-G), which is well in line with previous reports implicating the CDK2 axis downstream of CCNE1-mediated resistance to CDK4/6 inhibition.^50–52^ Together, these findings support a model in which sustained CDK activity, either CDK4/6 through cyclin D stabilization or CDK2 via interaction with cyclin E1, enables continued proliferation despite CDK4/6 inhibition.

Beyond the resistance-associated role of AMBRA1 during CDK4/6 inhibition, our data further indicate that AMBRA1 influences replication stress responses. AMBRA1 loss resulted in increased CHK1 levels, elevated endogenous replication stress, and enhanced dependency on the checkpoint pathway (Figure 4A-C). Consequently, AMBRA1-deficient cells were hypersensitive to CHK1 inhibition and displayed synergistic cytotoxicity when CHK1 inhibitors were combined with CDK4/6 inhibition. Mechanistically, our data suggest that this interaction occurs through CRL4-dependent regulation rather than AMBRA1’s canonical role in autophagy (Figure 4D-F). In addition, AMBRA1 deficiency sensitized cells to ionizing radiation, particularly during S/G2-M phases, indicating that impaired replication stress resolution creates therapeutic vulnerabilities that can be further exploited (Figure 4G-M).

Our CRISPR knockout screens identified *CHEK1* and *FAM122A* as synthetic lethal interactors to CDK4/6 inhibition in glioma cells (Figure 1A). Functional validation demonstrated that inhibition of *CHEK1* or *FAM122A* synergizes with CDK4/6 inhibition to induce cell-cycle arrest and apoptosis (Figures 5 and 6). *CHEK1* inhibition likely potentiates CDK4/6 inhibitor activity by exacerbating replication stress and activating ATR and p53-mediated DNA damage signaling, whereas *FAM122A* loss may modulate PP2A-dependent signaling networks involved in DNA damage responses (Figure 5E-H). These perturbations ultimately converge on cell-cycle regulation, resulting in reduced RB phosphorylation and decreased CDK2 activity during CDK4/6 inhibition. Additionally, combined inhibition of CDK4/6 and CHK1 produced synergistic antitumor effects *in vivo* in an LN229 xenograft model (Figure 6G) and in *ex vivo* cultures derived from primary human glioblastoma samples (Figure 6F). These data are consistent with studies demonstrating enhanced efficacy of combined CDK4/6 and checkpoint kinase inhibition in other cancer models.^53^^,^^54^ Furthermore, publicly available single-cell RNAseq data^37^^,^^38^ displayed high expression of *CHEK1* in tumor cells (Figures S33-S36), indicating potential context-dependent therapeutic vulnerabilities.

In summary (see Graphical Abstract; Figure 6I), our findings identify *AMBRA1* loss and *CCNE1* activation as two distinct mechanisms that reduce sensitivity to CDK4/6 inhibitors, with *AMBRA1* loss acting through CDK4/6-D-type cyclins and *CCNE1* activation through CDK2/Cyclin E pathways, highlighting dysregulation of cell-cycle control. Synthetic lethal interactions identified through our ­knockout screens revealed DNA damage response vulnerabilities involving *CHEK1* and *FAM122A* that can be therapeutically exploited in combination with CDK4/6i. Importantly, while CDK4/6i resistance mechanisms have been described in other cancer types, their relevance in glioma has not yet been established. Our study not only elucidates the molecular basis of CDK4/6 inhibitor resistance in glioblastoma but also identifies rational combination strategies to overcome this resistance. Our findings establish a mechanistic link between cell-cycle dysregulation and checkpoint dependency in experimental glioma and support future exploration of potential biomarker-driven combination therapies aimed at enhancing CDK4/6 inhibitor efficacy.

## Supplementary Material

noag093_Supplementary_Data

## Data Availability

All data from this study are publicly available. Raw data from CRISPR-Cas9 screens can be found at figshare (https://doi.org/10.6084/m9.figshare.29569775.v1). RNA sequencing data can be found at GEO under GSE247498 and GSE303100 (for review purposes, please use access token **cdozcaiipzwnxeb**). DNA methylation array data from experimental glioma cell lines can be found at GEO (GSE320594). Exome sequencing data from glioma cell lines have been deposited at SRA (BioProject ID PRJNA1425258). For reviewing purposes, use the following tokens: For EPIC arrays https://www.ncbi.nlm.nih.gov/geo/query/acc.cgi?acc=GSE320594 Enter token qnkzikkillqbtqz into the box. For exome sequencing: https://dataview.ncbi.nlm.nih.gov/object/PRJNA1425258?reviewer=ufvv6cf5fbltc6425ukbc5rcmu Further details for all methods are outlined in Supplementary Material S1.
