# Supplementary material for: Molecular modulators of cyclin-dependent kinase 4/6 inhibitor response in experimental glioma identified through genome-wide CRISPR-Cas9 screening
Source: Neuro Oncol. 2026 May 4;28(8):1904–20. doi: 10.1093/neuonc/noag093 (PMC13437906; doi:10.1093/neuonc/noag093)
Supplement: Supplementary file 1 [file noag093_supplementary_data.zip › N-O-D-25-01231R1_Supplementary info_CLEAN.docx]

**Molecular modulators of CDK4/6 inhibitor response in experimental glioma identified through genome-wide CRISPR-Cas9 screening**

Surender Surender, Lara Annina Haeusser, Laurence Kuhlburger, Foteini Tsiami, Nihal Olcay Dogan, Louise Maise, Sven Nahnsen, Susanne Beck, Daniel Josef Merk, and Ghazaleh Tabatabai^*^

**Supplementary Information**

**Content:**

- Supplementary Methods
- Supplementary Figures S1 – S36
- Supplementary Table S1
- Supplementary References

### Supplementary Methods

### RNA Sequencing

Glioma cell lines LN229, LN229 sgAMBRA1, LN18, LN308, T98G, GS-2, and GS-9 were seeded at their optimal density and treated the next day with either abemcaiclib or their respective DMSO controls for 48h. RNA isolation was performed using the RNeasy Plus Mini Kit (Qiagen, Venlo, NL) according to the manufacturer’s protocol. mRNA fraction enrichment was done with polyA capture from 200 ng of total RNA using the NEBNext Poly(A) mRNA Magnetic Isolation Module (NEB). For the preparation of mRNA libraries, we employed the NEB Next Ultra II Directional RNA Library Prep Kit for Illumina (NEB), following the manufacturer’s instructions. Sequencing was carried out on an Illumina NovaSeq 6000 system (Illumina, San Diego, CA, USA) using a paired-end format with 50 bp read length, achieving a depth of approximately 25 million clusters per sample.

Bioinformatic processing and data management were conducted at QBiC, Tübingen, Germany. RNA-seq analysis was performed using the nf-core/rnaseq pipeline (version 1.4.2; https://github.com/nf-core/rnaseq, accessed on 21 January 2020), which is implemented with Nextflow. As part of this workflow, FastQC (v0.11.8) was used to assess the initial quality of the FASTQ files. Adapter and quality trimming were performed using Trim Galore (v0.6.4). Reads passing quality control were mapped to the GRCh37 reference genome using the STAR aligner (v2.6.1). Additional RNA-seq quality control metrics were assessed with RSeQC (v3.0.1), and read counting was handled using featureCounts (v1.6.4). Finally, a comprehensive summary of quality control results was generated using MultiQC (v1.7; http://multiqc.info/, accessed on 21 January 2021). Further details can be found under https://nf-core.re/rnaseq.

Differential gene expression analysis was performed using the DESeq2 R package. We first normalized the read counts using the median of ratios method from DESeq2 . We used factoextra and stats R packages to perform principal component analysis. The exclusion criteria for genes were based on the presence of normalized read counts in at least three samples. Significantly differentiated expressed genes (DEGs) were determined by setting a threshold of |log2fold change| ≥ 0.58 and padjusted value < 0.05. We applied the Wald statistics to get DEGs between conditions, such as DMSO or abemaciclib, using a “design = ~condition” formula. We then used a “design = ~cell_line + condition” formula to determine common changes under abemaciclib treatment across all cell lines to eliminate any gene expression changes linked to distinct cellular backgrounds. To assess differential gene expression in response to abemaciclib treatment across all pairwise combinations of cell lines, we employed a linear interaction model using the formula, “design = ~ cell_line + condition + cell_line:condition” in DESeq2. Interaction terms were extracted from the model using Wald statistics to identify genes with treatment effects that differed between cell lines. The resulting set of candidate interaction genes was subsequently clustered based on expression patterns using the degPatterns function from the DEGreport R package.

### Single-cell RNA Expression Datasets

We analyzed two publicly available and pre-processed single-cell RNA sequencing (scRNA-seq) datasets obtained from the Single Cell Portal.^1^ The first dataset comprised 16 IDH–wild-type glioblastoma (CNS WHO grade 4) samples, totaling 182,330 cells.^2^ Pediatric brain tumors and low-grade gliomas were excluded from subsequent analyses. Tumor cells were annotated according to the four principal cellular states defined in the second dataset^3^, which included 20 tumor samples encompassing 5,742 cells. Pediatric high-grade glioma samples were again excluded to ensure consistency across datasets. All malignant cells were classified into the four previously defined cellular states based on available metadata. To normalize for differences in cell population size, we calculated the proportion of cells within this upper percentile relative to the total number of cells per cell type, yielding a normalized measure of marker gene enrichment across populations. Error bars represent the standard error across all included patient samples.

### Copy number variant analysis using EPIC arrays

### Genomic DNA was extracted from glioma cell lines using FFPE material using the QIAamp DNA Mini and Blood Mini kit from Qiagen, the Netherlands. DNA was processed on the MethylationEPIC BeadChip (Illumina) according to the manufacturer’s instructions at the Microarray Core Facility at DKFZ Heidelberg. The openSesame pipeline from the SeSAMe package was used to generate methylation beta values. CNV profiles from DNA methylation data were generated using the conumee2 package. RB1 pathway-associated genes were predefined, and CNVs potentially affecting these loci were interrogated using the CNV.focal function from conumee2.0 using a confidence level of 0.99 to determine the log2-threshold for focal alterations.

### Exome sequencing

Primary data analysis starting from RAW sequencing data to annotated variant files was carried out using the Nextflow workflow Sarek v2.7, including quality control, read mapping, and variant calling. After adapter trimming, sequencing reads were aligned to the human reference genome GRCh38 with BWA v0.7.17-r1188 (Li and Durbin, 2009). For variant calling, the aligned reads were processed to identify SNPs, MNPs (multiple nucleotide polymorphisms, i.e., adjacent SNPs), and Indels. As part of the secondary analysis, SnpEff-annotated variant files were filtered to retain only protein-coding variants that were either missense or stop-gain mutations. Subsequently, vcfstats v1.1 was used to quantify the number of SNPs per chromosome for each sample. The resulting filtered and annotated variants are summarized in Supplementary Table 2.

### Acute Cytotox Assays

We seeded 5×10^3 or 10×10^3 cells per well in 96-well flat-bottom plates on day 1, followed by the indicated treatments on day 2 and incubation for 72 h. Cell viability was assessed using CellTiterBlue reagent (Promega, Madison, WI, US) utilizing a GloMax (Promega, Madison, WI, US) as previously described.

### Synergy Assays

Cells were treated for 72 hours in a 4x4 concentration matrix covering single drug treatments and combinations of both drugs. DMSO controls were used for data normalization. Cell viability was measured using the CellTiter-Blue reagent on the GloMax. Zero interaction potency model (ZIP) comprising the *synergyfinder* R package^25^ was used to calculate synergism scores based on cell viability. Synergy analysis was performed to determine whether the effect exceeded simple additivity. Using the ZIP model, we applied a predefined threshold: a mean score>10 indicates synergism, scores between -10 and 10 indicate additive effects, and scores <-10 indicate antagonism. When described as synergistic, observed responses were greater than the predicted additive responses within specific dose regions, indicating a synergistic interaction rather than purely additive effects.

### Clonogenic Survival Assays

We seeded 250 0r 750 cells per well on day 1, treated the cells with the indicated concentrations on day 2. After 24h of incubation, we replaced the media with serum-containing media and let the cells grow for 14 to 21 days. The colonies were stained with0.5% w/v crystal violet and counted manually. Data were normalized to respective vehicle control wells.

### Cell Cycle Analysis

Cells were seeded at their respective optimal density one day prior to the treatment. Afterwards, 48h of treatment either with abemaciclib or DMSO control was conducted. Then the cells were detached from the flask and washed with sample buffer (PBS (phosphate-buffered saline) (Gibco, Carlsbad, CA, US) containing 1 g/L glucose (Roth, Karlsruhe, Germany). Cells were fixed in 80% ice cold ethanol and incubated for 15 min at −20 °C. Cells were rehydrated in the sample buffer for 15 min at room temperature. Afterward, the cells were stained for 15 min at room temperature in staining solution (50 μg mL−1 propidium iodide (Thermo Fisher Scientific, Waltham, MA, US), 0,2% Triton X-100 (Roth, Karlsruhe, Germany), 100 μg Rnase (Thermo Fisher Scientific, Waltham, MA, US), sample buffer) and they were analyzed using a MACSQuant Analyzer 10 (Miltenyi Biotec, Bergisch Gladbach, Germany). A minimum of 100,000 events were recorded for proper gating. The acquired data were analyzed using FlowJo software (BD, Franklin Lakes, NJ, US).

### BrdU Incorporation Assays

We seeded glioma cells at day 0 and treated them with either DMSO control or abemaciclib the next day. 24h or 48h post tretament, the cells were given a short pulse of 45 minutes with 10 µM BrdU (BD Biosciences). Cells were washed with PBS and fixed in 70% ethanol (-20°C, overnight) for permeabilization. Next morning, cells were washed with PBS and denaturation was performed using 2N HCl (30 min at RT, in the dark), followed by neutralization with 0.1M sodium borate buffer (pH 8.5, 10 min). After a washing step with PBS + 0.5% BSA + 0.1% Tween-20, cells were incubated with anti-BrdU antibody (diluted in 1:50–1:200 in PBS-BSA-Tween, 30 min at RT), washed with PBS, and stained with 1x propidium iodide containing RNAseA. The data were acquired using a MACSQuant Analyzer 10 (Miltenyi Biotec, Bergisch Gladbach, Germany) and analyzed with the FlowJo software (BD, Franklin Lakes, NJ, US).

### Apoptosis Analysis

We used the Apoptosis BD detection Kit (BD Biosciences, Heidelberg, Germany). In brief, cells were detached and washed with PBS, followed by incubation in the staining solution (containing 5µl Annexin V and 5µl propidium iodide in 1x binding buffer) for 15 min at RT in the dark. Single-stained controls or FMOs (Fluorescence minus one) were used for proper gating. The data were acquired using a MACSQuant Analyzer 10 (Miltenyi Biotec, Bergisch Gladbach, Germany) and analyzed with the FlowJo software (BD, Franklin Lakes, NJ, US).

### Immunocytochemistry

LN229 parental or LN229 sgAMBRA1 cells (50,000 per well) were seeded on poly-L-lysine–coated Permanox 8-chamber slides (Thermo Fisher Scientific, Waltham, MA, US). Four hours after seeding, cells were treated as indicated. After 24 h, cells were fixed with 4% paraformaldehyde (PFA), washed with PBS, and permeabilized with 0.2% Triton X-100 (Carl Roth GmbH, Karlsruhe, Germany). After additional PBS washes, cells were blocked with 2% bovine serum albumin (BSA) and incubated overnight at 4 °C with primary antibodies against γH2AX and cleaved caspase 3 (Cell Signaling Technology, Danvers, MA, US), diluted in 0.1% BSA.

Following PBS washes, cells were incubated for 1h at room temperature with Alexa Fluor 555- or 594-conjugated donkey anti-rabbit secondary antibodies (Thermo Fisher Scientific, Waltham, MA, US or Jackson ImmunoResearch, Baltimore, US; 1:500), followed by nuclear counterstaining with DAPI (Thermo Fisher Scientific, Waltham, MA, US) (1:2500 in 0.1% BSA) for 10 min in the dark. After final washes with PBS-T, chambers were removed, and slides were mounted using Fluoromount aqueous mounting medium (Sigma Aldrich, St. Louis, MO, US). Images were acquired on a Cytation C10 confocal imaging reader (Agilent, Santa Clara, US) using a 20x or 40x objective and a 40 mm pinhole disk. Z-stacks (15 optical sections, 0.5 μm step size) were captured, and maximum intensity projection (MIP) images were generated using Gen5 software (Agilent, Santa Clara, US). No signal was detected in secondary antibody-only controls.

### ICC-based cytometry

Cells were grown on glass slides according in serum containing medium. 24h later, they were fixed in 4% paraformaldehyde in PBS for 15 min and washed 3 times in PBS. Next, permeabilization was performed in PBS plus 0.2 % Triton X-100 for 5 min at room temperature, followed by 1h of blocking in the blocking in 1% BSA. The slides were then incubated with primary antibodies in 0.1% BSA blocking buffer for 1 hour at RT and washed with PBS containing 0.1 % Triton X-100.

Cells were incubated with secondary antibodies (donkey anti-rabbit IgG (H+L) Alexa Fluor 555, Invitrogen, 1:500; goat anti-chicken IgY (H+L) Alexa Fluor 647, Jackson ImmunoResearch, 1:500) for 1h and washed. DNA was stained using propidium iodide for 10 min at RT, washed, and mounted. Images of fluorescently-labeled cells were acquired using a spinning disk confocal microscope (Cytation C10, BioTek), and the quantification of positive signal was done with the cellular analysis mode of Gen5 software (version 3.16, BioTek). For each condition, we used 3 independent experiments, acquiring at least 900-1000 cells. PI nuclear staining was used to identify and detect γH2AX nuclear intensity through the EdU staining. Scatter plots were generated in R, illustrating γH2AX intensity as high or low across the EdU and PI stainings on the y- and x-axes, respectively.

### CDK2-Cyclin E1 kinase assays

We used a custom CDK2-Cyclin E1kinase activity kit from AssayQuant, United States. Glioma cells were seeded into T75 flasks in serum-containing medium. 24h later, cells were incubated with 2.5mM Thymidine for a 24h pulse. After 24h, the medium was replaced with fresh medium for 4h as a first release step and then incubated with Thymidine for a second pulse. Cell lysates were prepared after 48h in Cell extraction Buffer with protease (and not phosphatase) inhibitors (50mM HEPES, pH 7.4; 150 mM NaCl; 2mM EGTA; 1mM DTT; 1% Triton X-100; Protease inhibitor Cocktail diluted 60 fold). All lysates were freshly prepared and used right away to avoid any activity loss. Reactions were set up according to the manufacturer's protocol. 2.0 μg/well NIH-OVCAR3 crude cell lysate from NIH-OVCAR3 cells was used a positive control synchronized to G1/S Phase of cell growth. Briefly, 2.5 μl 10X AQT1170 Substrate dilutions were prepared 17.5μL Reaction Mix with ATP & DTT, and 2μg of cell lysates to a final volume of 25μL in a 96-well flat-bottom plate. The plate was sealed using a PCR film and incubated at 30 °C for 15 minutes to equilibrate first. Continuous kinetic measurements were performed on a GloMax Promega device reader with excitation at 360 nm and emission at 485 nm. The program was run for at least 240 minutes, with continuous readings and shaking at 30 °C at a rate of 1/5 minutes. Data were analysed in GraphPad Prism version 11.0.0.

### Immunoblot

Protein lysates were extracted from cells using Pierce RIPA buffer (Thermo Fisher Scientific, Waltham, MA, US) with phosphatase inhibitors cocktail (1:100) (#5870, Cell Signaling Technology, Danvers, MA, US), followed by sonication to ensure DNA shearing. 30µg-40µg of protein lysates were mixed with 4x LDS sample buffer and incubated at 70°C for 10 mins. 4-12% NuPage precast gels (Thermo Fisher Scientific) were used for electrophoresis, and blotting was done on nitrocellulose membranes. Membranes were blocked in 5% nonfat dry milk-TBST buffer for 1 h at RT and probed with primary antibodies overnight at 4°C. The next day, the membranes were probed with secondary goat anti-rabbit (1:5000, ab97051, Abcam, Cambridge, UK) or goat anti-mouse (1:5000, ab97023, Abcam, Cambridge, UK) horse radish peroxidase (HRP)-conjugated antibodies. Protein bands were visualized using ECL solution or SuperSignal West Dura solution (Thermo Fisher Scientific, Waltham, MA, US), and images were acquired in a ChemiDoc imaging machine (Bio-Rad Laboratories, Hercules, CA, USA).

We used the following primary antibodies (Cell Signaling Technology, Danvers, MA, US): AMBRA1(#24907, Cell Signaling), CCNE1 (#20808, Cell Signaling), Vinculin (#13901, Cell Signaling), GAPDH (#2118, Cell Signaling), Beta tubulin (#2146, Cell Signaling), c-MYC (#5605, Cell Signaling), RB (#9309, Cell Signaling), pRB (#8516, Cell Signaling), CCND1 (#55506, Cell Signaling), CCND2 (#3741, Cell Signaling), CCND3 (#2936, Cell Signaling), AURKB (#28711, Cell Signaling), CDK1 (#14866, Cell Signaling), CDK2 (#2546, Cell Signaling), CDK4 (#12790, Cell Signaling), CDK6 (#13331, Cell Signaling), mTOR (#2972, Cell Signaling), phospho mTOR (#2971, Cell Signaling), FAM122A (#24179, Cell Signaling), CHK1 (#2360, Cell Signaling), p27 (#3698, Cell Signaling), pCHK1 (#12302, Cell Signaling), γH2AX (#9718, Cell Signaling), Cleaved PARP (#5625, Cell Signaling), PARP (#9542, Cell Signaling), ATM (#2873, Cell Signaling), pATM (#5883, Cell Signaling), AKT (#9272, Cell Signaling), pAKT (#4060, Cell Signaling), ATR (#2790, Cell Signaling), DNMT1 (#5032, Cell Signaling), FOXM1 (#20459, Cell Signaling), pFOXM1 (#14170, Cell Signaling), pCDK1 (pCDC2, Tyr15) (#4539, Cell Signaling), CHK1 (#37010, Cell Signaling), WEE1 (#13084, Cell Signaling), PPP2R2A (#16569-1-AP, ProteinTech), CDC25A (#sc-7389, Santa Cruz), DDB1 (#6998, Cell Signaling), ATR (#13934, Cell Signaling), p53 (#2524, Cell Signaling), FLAG (#F7425, Sigma-Aldrich).

### Co-immunoprecipitation

FLAG and FLAG-AMBRA1 cells were used to prepare lysates using the Pierce IP lysis buffer, Thermo Scientific, MA, United States. For co-immunoprecipitations, we used the Anti-Flag M2 magnetic beads from Millipore, MA, United States. Briefly, a similar amount of total cellular lysates was loaded on slurry mixed anti-Flag M2 magnetic beads. Following an overnight incubation at 4 °C in an end-over-end shaker, the supernatants were discarded, and the beads were washed twice with Tris Buffer (pH 7.4). The protein was eluted for SDS-PAGE using the sample buffer used in immunoblotting, by boiling the mix at 95 °C for 3 minutes. Beads were collected using a magnetic separator, and the samples were loaded onto the SDS-PAGE gels followed by immunoblotting using nitrocellulose membranes.

### Alpha fold prediction

We used the amino acid sequence of the AMBRA1WD40 domain, along with full-length sequences of selected cell cycle regulators, to analyze heteromeric protein complexes. We modeled the protein complexes using ColabFold (v1.5.2) from GitHub (https://github.com/sokrypton/ColabFold), which implements the AlphaFold2 algorithm combined with MMseqs2 for multiple sequence alignment. For complex prediction, we used the alphafold_multimer_v3 model with 20 recycling iterations. The protein data bank file from the highest-ranking model was imported into ChimeraX to generate protein cartoon representations. The corresponding .json files were used to visualize inter-chain AlphaFold contacts within an eight-angstrom cutoff. Predicted alignment error matrices for all pairwise residue comparisons in heteromeric complexes, as well as predicted template modeling scores, were plotted using a custom Jupyter notebook.

### Glioma Cell Lines and Glioma Stem-Like Cells

The glioma cell lines LN18, LN229, LNZ308, and T98G were maintained in Dulbecco’s Modified Eagle Medium (DMEM; Gibco, Carlsbad, CA, USA) supplemented with 10% fetal calf serum (FCS; Thermo Fisher Scientific, Waltham, MA, USA) and 50 µg/mL gentamicin (Thermo Fisher Scientific, Waltham, MA, US). Cell line authentication was performed through the Leibniz Institute DSMZ–German Collection of Microorganisms and Cell Cultures GmbH.

Glioma stem-like cells, GS-2 and GS-9, were cultured in Neurobasal®-A medium (Gibco, Carlsbad, CA, US), enriched with 2% B27 supplement without vitamin A (Invitrogen, Waltham, MA, USA), 1% GlutaMAX (Thermo Fisher Scientific, Waltham, MA, US), and 0.02 µg/mL each of recombinant human fibroblast growth factor (FGF) and epidermal growth factor (EGF) (PeproTech, Cranbury, NJ, USA). All cell lines were cultured under standard conditions at 37 °C in a humidified atmosphere containing 5% CO₂.

### Primary Glioblastoma Cultures

Primary glioblastoma cell cultures were generated from freshly resected tumor tissue at the Department of Neurosurgery, University Hospital Tübingen, as previously described (ethics approval 225/2023 BO2). In brief, tissue was minced, enzymatically dissociated with collagenase and dispase (Roche, Basel, CH), and erythrocytes were removed using lysis buffer (Sigma Aldrich, St. Louis, MO, US). Cultures were maintained under the same conditions as GS neurospheres. Drug treatments and assessment of synergy using the Bliss Independence model followed established protocols.

### Patient-Derived Microtumors

Primary glioblastoma tissue was washed with 5 mL HBSS, mechanically minced in a culture dish, and transferred to 20 mL HBSS (Gibco, Carlsbad, CA, US)^4^. The resulting suspension was centrifuged at 200 × g for 5 minutes at 4°C. The pellet was resuspended in 20 mL DMEM containing 1% penicillin-streptomycin, transferred to a 125 mL conical flask, and digested with Liberase at 37°C for 2 hours on an orbital shaker. After centrifugation (300 × g, 5 min, 4°C), the pellet was resuspended in 20 mL HBSS, filtered sequentially through 500 μm and 40 μm strainers, and washed with HBSS. Microtumor fragments were cultured in suspension in 4 mL of Neurobasal medium with FGF and EGF in 6 cm dishes. For cryopreservation, microtumors were frozen in 70% culture medium, 20% FCS, and 10% DMSO.

### Orthotopic Murine Models

Animal experiments were approved through the Tübingen regional council under the license number N04/21G and conducted in compliance with animal welfare regulations. 6 to 7-week-old female CD1 nude mice were purchased from Charles River Laboratories (Sulzfeld, Germany) and were routinely analysed for infectious diseases. In line with the previously described procedures, we injected 75,000 LN229 human glioma cells into the right striatum of the 8-9-week-old mice. The mice were randomized into four treatment groups (n=9 per group) and received abemaciclib (MedChemExpress LLC, Monmouth Junction, NJ, US) monotherapy, AZD7762 (MedChemExpress LLC, Monmouth Junction, NJ, US) monotherapy, a combination of both drugs, or vehicle control. Abemaciclib was administered orally at 75mg/kg bodyweight and AZD7762 at 20mg/kg bodyweight intraperitonially. We started the treatments at day 8 post-surgery and continued for a total of 3 weeks (5 days per week)(Figure 6G). Animals were closely monitored following the regulated score sheets (Supplementary Table S1). Experimental endpoints were defined according to animal law, “time until the onset of neurological symptoms” referred to as “survival,” and the experiments were ended on day 120.

### Histological and Immunohistochemical Analysis of Post-Treatment Brains

Animal brains were harvested at experimental endpoints, snap frozen in liquid nitrogen, and fixed in Tissue-Tek medium (Sakura Finetek Europe B.V., Alphen aan den Rijn, Netherlands). Sections of 8 µm thickness were cut using a cryotome (LEICA CM3050S) (Leica, Wetzlar, Germany) and stored at -80°C. Hematoxylin (Sigma-Aldrich, St. Louis, MO, US) and Eosin (Roth, Karlsruhe, Germany) (H&E) staining was performed as described previously. Immunohistochemistry was performed by fixation of murine brain sections either in 4% PFA (RT) or acetone (-20 °C) and 80% methanol (4°C), based on the type of primary antibody used. After a blocking step in 2% BSA in PBS-Tween 0.06%, the slides were incubated with the primary antibody. We used Ki67 (1:100, ab16667, Abcam, Cambridge, UK), Cleaved PARP (1:100, #5625, Cell Signaling, Frankfurt am Main, Germany), CHK1 (1:100, #2360, Cell Signaling, Frankfurt am Main, Germany), and γH2AX (1:100, #9718, Cell Signaling, Frankfurt am Main, Germany). The next day, the sections were incubated with secondary antibodies followed by incubation with avidin/biotin-based peroxidase solution (VECTASTAIN Elite ABC, Vector Laboratories, Peterborough, UK) for 30 minutes. Finally, the sections were stained with NovaRed Substrate-HRP solution (Vector Laboratories, Burlingame, CA, US) for 1-5 minutes and counterstained with Hematoxylin for 45 seconds. Images were acquired using bright-field microscopy (Zeiss, Axioplan 2, Carl Zeiss, Oberkochen, Germany ) and analyzed on Adobe Photoshop CS5.1 and Gimp (2.10.34).

### Supplementary Figure S1

**Supplementary Fig.S1: CRISPR-Cas9 screens reveal genetic modifiers of CDK4/6 inhibition response in human glioma models.** (A) Schematic representation of the activation/knockout screen model. Created in BioRender (<https://BioRender.com/tgrlp61> ). **(B**) Cell viability post 72h of abemaciclib treatment in long-term glioma cell lines (LN229, LN18, LNZ308, T98G) and stem-like cells (GS-2, GS-9) measured via acute cytotox assays; normalized to DMSO controls. (C) Copy number variation analysis of both long-term (LN229, LN18, LNZ308, and T98G) and stem-like (GS-2 and GS-9) glioma models.

### Supplementary Figure S2

**Supplementary Fig.S2: Molecular backgrounds of glioma models**. (A-C) RNA sequencing analysis of glioma cell lines. (D) Immunoblots for RB1 pathway-associated proteins. (E) Normalized mRNA counts across all cell lines from bulk RNAseq. Blot images are representative of n=3.

### Supplementary Figure S3

**Supplementary Fig.S3: Molecular backgrounds of glioma models**. Uncropped western blots for Supp. Fig. S2D

### Supplementary Figure S4

**Supplementary Fig.S4: CRISPR-Cas9 screens reveal genetic modifiers of CDK4/6 inhibition response in human glioma models**. (A) Scatter plots illustrating simulated screen Beta values from a drug and a vehicle screen compared to the plasmid library. Screen hits involved in drug response can be identified by their deviation from the linear regression line. (B) Scatter plots and Rank view plots illustrating comparison of drug vs DMSO-treated samples; Activation screens LNZ308. (C) Hallmark gene set enrichment analysis of drug-specific, uniformly scoring, positively selected hits from the knockout screens, i.e., resistance candidate genes. (D) Hallmark gene set enrichment analysis of drug-specific, uniformly scoring, negatively selected hits from the activation screens. i.e., synthetic lethal candidate genes.

### Supplementary Figure S5

**Supplementary Fig.S5: CRISPR-Cas9 screens reveal genetic modifiers of CDK4/6 inhibition response in human glioma models. Quality control analysis of CRISPR-Cas9 screens.** (A, B) Volcano plots of DMSO arms from GS9 and LN229 knockout screens, highlighting depletion of known essential genes (red). (C) GS9-DMSO sample showing adjusted distribution after correction using CRISPRcleanR. (D) Gene-level depletion profile for GS-9 DMSO sample after imposing pan-essential gene sets; recall at 5% false discovery rate (FDR) is indicated in blue. (E) Copy number variation level correction in the GS-9 DMSO sample. (F) Normalized read counts in the GS-9 sample. (G) Gene set enrichment analysis (GSEA) of GS-9 DMSO samples showing depletion of pan-essential gene sets as identified by MAGeCK maximum likelihood distribution (MLE).

**Supplementary Figure S6**

**Supplementary Fig.S6: Functional validation of CRISPR-Cas9 screen hit.** (A) Guide RNA mediated knockouts of AMBRA1 and overexpression of CCNE1 were performed in human glioma cell lines. (B) Uncropped blots of western validation of generated genetic constructs in LN229 and GS-9 cells; GAPDH was used as housekeeping control. (C-D) Immunoblots showing expression of cell cycle-associated proteins in parental vs *AMBRA1* knockout cells, LN229, LN18, LNZ308, and GS-9. GAPDH was used as a loading control. Images are representative of n=3.

**Supplementary Figure S7**

**Supplementary Fig.S7: Influence of *AMBRA1* depletion on cell cycle-associated proteins**. Full uncropped western blots Supplementary Fig. S6C-D. Images are representative of n=3.

**Supplementary Figure S8**

**Supplementary Fig.S8: CDK4/6 inhibitor sensitivity in glioma cell lines and further validation of resistance hits** (A) Schematic representation of flow cytometry-based assessment of cell cycle using PI staining in LN229 and LN18 cell lines; cells were treated with either DMSO or abemaciclib for 24h. (B) Schematic representation of a flow cytometry-based gating scheme for cell cycle analysis.

**Supplementary Figure S9**

**Supplementary Fig.S9: Dose-dependent apoptotic response to abemaciclib treatment.** (A, B) Schematic representation of flow cytometry-based assessment of apoptosis using annexin V vs PI staining in parental vs sgAMBRA1 vs CCNE1oe constructs of LN229 and GS-9 cell lines; cells were treated with either DMSO or abemaciclib for 48h. (C, D) Quantification of early apoptotic (Annexin high, PI low) events in LN229 (left) and GS-9 (right) cells. (E, F) Quantification of late apoptotic (Annexin high, PI high) events in LN229 (left) and GS-9 (right) cells. Statistical analysis: two-way ANOVA, *padjust < 0.05, ** padjust < 0.01, only significant differences are displayed. All experiments were performed in biological triplicates unless otherwise indicated. Error bars represent mean ± SD.

**Supplementary Figure S10**

**Supplementary Fig.S10: Differential protein regulation under CDK4/6i in glioma lines**. Immunoblots illustrating cell line-specific expression of proteins associated with the RB1 pathway in glioma cell lines (LN229, LN18, and LNZ308) under increasing dosages of abemaciclib. Images are representative of n=3.

**Supplementary Figure S11**

**Supplementary Fig.S11: Response to CDK4/6 inhibition upon *AMBRA1* depletion and *CCNE1* overexpression**. (A) Western blot analysis of cell cycle regulators (CDK2, CDK4, pRB, mTOR, pmTOR, and p27) expression in parental vs sgAMBRA1 vs CCNE1oe LN229 cells under increasing doses of abemaciclib. Beta tubulin was used as a loading control. (B) Full uncropped blots from (A). Images are representative of n=3.

**Supplementary Figure S12**

**Supplementary Fig.S12: Distinct activity of *AMBRA1* loss and *CCNE1* gain-of-function in glioma cells.** (A) Full uncropped westerns from Figure 2E. (B) CDK2/Cyclin E1 kinase activity curves from LN229 cell lysates. RFU indicates relative fluorescence unit measured over 240 minutes and corrected to control, comparing all three constructs under basal (left) and CDK2i (right) treated conditions. Blot images are representative of n=3.

**Supplementary Figure S13**

**Supplementary Fig.S13: Modified transcriptional changes under CDK4/6 inhibition upon *AMBRA1* depletion**. (A) Pair-wise correlation of transcriptome from LN229 parental and sgAMBRA1 cells treated with abemaciclib or DMSO (vehicle) for 48 hours; inclusive of individual replicates. (B) Volcano plots of differential gene expression in parental (left) and sgAMBRA1 (right) cells following abemaciclib treatment. Genes significantly downregulated across all cell lines (log2FC < -1, padj < 0.05) are highlighted in blue, and commonly upregulated genes (log2FC > -1, padj < 0.05) in red. (C) KEGG, Hallmark, and Reactome gene set analysis from interaction genes. (D) 9-square plot comparing differential expression of sgAMBRA1 (y-axis) vs parental (x-axis) cells under abemaciclib treatment. (E-G) Hallmark gene set enrichment analysis of genes as defined in groups from the 9-square comparison plot.

**Supplementary Figure S14**

**Supplementary Fig.S14: Modified response to CDK4/6 inhibition upon *AMBRA1* depletion.** (A) Full uncropped western blots to Figure 1H. (B) Schematic representation of a flow cytometry-based gating scheme for cell cycle analysis. (C,D) BrdU incorporation in LNZ308 cells treated with abemaciclib.

**Supplementary Figure S15**

**Supplementary Fig.S15: AMBRA1 loss remodels gene expression upon CDK4/6 inhibition:** (A) Western validation of *MYC* knockout and *MYC* overexpressing LN229 cells. (B) Dose response curves illustrating the viability of GS-9 cell lines in parental, MYC knockout, or MYC overexpressing conditions under 72h abemaciclib treatment. (C) Quantification of % LN229 cells in G2-M phase (left), flow cytometry-based cell cycle analysis of parental vs sgMYC LN18 cells either treated with DMSO, abemaciclib, or pevonedistat plus abemaciclib for 24h (right). Blot images are representative of n=3.

**Supplementary Figure S16**

**Supplementary Fig.S16: AMBRA1 loss sensitizes glioma cells to CHK1 inhibition.** (A) 3 Dimensional synergy maps showcasing potential synergy between abemaciclib and CHIR-124, comparing parental cells AMBRA1 defficient cells. (B) Synergy heatmaps showing individual effects per paired combination as generated using Synergyfinder in R. (C) Dose response curves from abemaciclib treatment showing direct drug treatment effects. (D) Clonogenic survival assays comparing parental vs sgAMBRA1 cells under the indicated treatments.

**Supplementary Figure S17**

**Supplementary Fig.S17: AMBRA1 interacts with CHK1 in glioma cells:** (A) Uncropped blots to Figure 4A. (B-C) Complex structure prediction of AMBRA1WD40 with DDB1 and Cyclin D1 using AlphaFold2_mmseqs2. Top: cartoon representations of the predicted heteromeric protein complexes. Bottom: pairwise PAE scores for each protein complex. (D-E) Uncropped blots to Figure 4E-F.

**Supplementary Figure S18**

**Supplementary Fig.S18: AMBRA1 loss sensitizes glioma cells to ionizing radiation.** (A) Clonogenic survival under single dosages of irradiation. (B) Heatmaps illustrating cell viability of LN229 cells under single-dosage irradiation, measured after 24 or 48 hours. (C) Three-dimensional ZIP synergy plots showing the degree of synergy between irradiation and pevonedistat at 24h (top) or 48h (bottom) treatment. (D) Bar plots representing FC measured γH2AX intensity in parental vs sgAMBRA1 LN229 cells under increasing dosages of radiation.

**Supplementary Figure S19**

**Supplementary Fig.S19: AMBRA1 loss sensitizes glioma cells to ionizing radiation.** (A) Uncropped blots to Figure 4M. (B) Quantification of pATM and PARP normalized to housekeeping control showing expression under indicated treatments, comparing parental vs sgAMBRA1 cells. Images are representative of n=3.

**Supplementary Figure S20**

**Supplementary Fig.S20: Functional validation of synthetic lethal interactors.** (A, B) Full uncropped western blot analysis of CHEK1 (A) and FAM122A (B) knockouts generated using LN229 cells. Vinculin was used as a loading control. (C) Proliferation assays comparing knockouts of *FAM122A* and *CHEK1* in parental LN229 (top) and LN18 (bottom) cells. (D). BrdU incorporation in parental vs sgCHEK1 or sgFAM122A glioma cell lines. (E,F) Acute (72h) (D) and long-term (12 days) dose response curves illustrating viability of GS-9 (top) and LNZ308 (bottom) cell lines under abemaciclib treatment; normalized to DMSO controls. Statistical analysis: two-way ANOVA, *padjust < 0.05.

**Supplementary Figure S21**

**Supplementary Fig.S21: Modified response to CDK4/6 inhibition upon *CHEK1* or *FAM122A* depletion**. (A) Flow cytometry-based assessment of apoptosis using annexin V staining in parental, sgFAM122A, and sgCHEK1 LN18 cells; cells were treated with either DMSO (left) or abemaciclib (right) for 48h. (B) Schematic representation of flow cytometry-based assessment of cell cycle using PI staining parental, sgFAM122A, and sgCHEK1 LN18 cells; cells were treated with either DMSO or abemaciclib for 24h. (C, D) Percentage of cells in G1 phase under DMSO or abemaciclib treatment comparing parental vs CHEK1 or FAM122A knockouts in LN229 (C) and LN18 (D) cells. (E, F) Percentage of BrdU positive cells under DMSO or abemaciclib treatment (200nM) comparing parental vs *CHEK1* or *FAM122A* knockouts in LN229 (E) and LN18 (F) cells.Statistical analysis: two-way ANOVA, *padjust < 0.05, ** padjust < 0.01, *** padjust < 0.001, only significant differences are displayed.

**Supplementary Figure S22**

**Supplementary Fig.S22: Genetic depletion of FAM122A or CHEK1 sensitizes glioma cells to CDK4/6 inhibition**. Uncropped blots to Figure 5F. Images are representative of n=3.

**Supplementary Figure S23**

**Supplementary Fig.S23: Genetic depletion of FAM122A or CHEK1 sensitizes glioma cells to CDK4/6 inhibition**. (A) Immunoblot assays comparing cells with either genetically or pharmacologically inhibited CHK1, treated with DMSO or abemaciclib for 48h. (B) Immunoblot assays on parental vs sgFAM122A cells, either treated with DMSO or abemaciclib for 48h; vinculin serves as the loading control. Images are representative of n=3.

**Supplementary Figure S24**

**Supplementary Fig.S24: Inhibition of CHEK1 and FAM122A interactors (WEE1, PPP2R2A):** (A-B), Cell viability post 72h of adavosertib (WEE1 inhibitor) (A) and LB100 (PP2A/PPP2R2A inhibitor) (B) treatment in long-term glioma cell lines measured via acute cytotox assays. (C) Acute dose response to LB100 treatment in parental vs FAM122A knockout LN229 cells. (D) Acute dose response to abemaciclib treatment in parental vs PPP2R2A knockout LN229 cells. Cell viability in all cytotox assays was measured by normalizing to respective DMSO controls.

**Supplementary Figure S25**

**Supplementary Fig.S25: Inhibition of CHEK1 and FAM122A interactors (WEE1, PPP2R2A):** (A-B) FC measured cell cycle histograms illustrating cells treated with increasing abemacilcib concentrations. Cells were serum-starved for 2h (A) or 12h (B) before adding serum-containing media and respective treatment.

**Supplementary Figure S26**

**Supplementary Fig.S26: CCGA analysis of CHEK1 and FAM122A expression in human GB:** (A) Kaplan-Meier survival curves from the CCGA GB dataset representing high or low CHEK1 or FAM122A expression. (B) Correlation plot illustrating Pearson correlation between CHEK1 and FAM122A expression.

**Supplementary Figure S27**

**Supplementary Fig.S27:** **Response to CHK1 inhibition across glioma cell lines.** (A, B) Pharmacological inhibition of CHK1 using CHIR-124 (a specific CHK1 inhibitor; A) and AZD7762 (B) in glioma cell lines, as illustrated in the acute cytotoxicity assays; normalized to DMSO controls. All experiments were performed in biological triplicates unless otherwise indicated.

**Supplementary Figure S28**

**Supplementary Fig.S28: Combined inhibition of CDK4/6 and CHEK1 and transcriptional response to CDK4/6i in glioma models.** (A) 3D Zip synergy plots from T98G, LNZ308, and GS-2 cells. (B) Clonogenic survival assays showcasing LN18 (left) or LN229 (right) cells treated with control, abemaciclib, CHIR-124, or combination. (C) Pair-wise correlation of transcriptome from glioma cell lines (GS-9, LN18, LN229) treated with abemaciclib or DMSO (vehicle) for 48h, inclusive of individual replicates. (C) Scree plot (left) showing the proportion of variance explained via the first 10 principal components (PCs), and principal component analysis (PCA) PC1 vs. PC2, PC1 vs. PC3, and PC2 vs. PC3 (right) for all indicated samples. (E, F) Gene set enrichment analysis (GSEA) of commonly regulated genes across all three cell lines reveals significant enrichment of the oxidative phosphorylation pathway (E) and depletion of pathways linked to cell cycle control and DNA replication (F). (G) Volcano plots of differential gene expression in each cell line following abemaciclib treatment. Genes significantly downregulated across all cell lines (log2FC < -1, padj < 0.05) are highlighted in blue; commonly upregulated genes (log2FC > -1, padj < 0.05) are highlighted in red.

**Supplementary Figure S29**

**Supplementary Fig.S29: Analysis of cell line-specific transcriptional changes under abemaciclib.** (A) Cell line-dependent effects of abemaciclib were determined using an interaction model (cell_line: treatment). Differentially expressed genes from all possible pairwise comparisons for three cell lines were clustered by expression patterns across treatment groups. (B, C) Venn diagrams illustrating the overlap of either upregulated (B) or downregulated (C) differentially expressed genes (DEGs) under abemaciclib treatment across all three cell lines.

**Supplementary Figure S30**

**Supplementary Fig.S30: Varying CHK1 regulation under CDK4/6 inhibition across glioma models.** (A) Normalized gene counts of top interaction genes in GS-9, LN18, and LN229 cells treated with either DMSO or abemaciclib for 48h. (B) Uncropped western blots from GS-9, LN229, or LN18 cells either treated with DMSO or abemaciclib for 48h, displaying Chk1 expression. (C-D) 3D Zip synergy plots from LN229 cells either under simultaneous (C) or sequential (D)(first AZD7762, then abema) treatment with abemaciclib and AZD7762. Blot images are representative of n=3.

**Supplementary Figure S31**

**Supplementary Fig.S31: Combination of CDK4/6 and CHK1i synergistically reduces glioma cell viability:** (A) FC measured fraction of LN229 cells in G1, S, and G2-M phases treated with abemaciclib, CHIR-124, their simultaneous combination (Combi sim.), or sequential combination (Combi seq.) (B) Apoptosis analysis under indicated treatment conditions in LN229 cells.

**Supplementary Figure S32**

**Supplementary Fig.S32: *Ex vivo* validation of synergism using primary human GBM cultures.** (A) Clinical and molecular features of primary human tumors. (B) Bar graphs representing viability of primary cultures under indicated treatment conditions normalized to DMSO controls. (B, C, D) Apoptosis analysis from primary culture TUE-PC16 under indicated mono or combination treatments; early apoptosis (C), late apoptosis (D). Statistical analysis: two-way ANOVA, *padjust < 0.05, ** padjust < 0.01, only significant differences are displayed. All experiments were performed in technical triplicates unless otherwise indicated (we managed to achieve three biological replicated for TUE-PC16). Error bars represent mean ± SD.

**Supplementary Figure S33**

**Supplementary Fig. S33: Quantitative analysis of single-cell RNA expression profiles of abemaciclib targets and DDR-associated proteins in publicly available data set (Neftel et al., Cell 2019**^3^**)**. Bar plots depict the fraction of cells within the top 10^th^ percentile for each target. Dots are indicative of values from individual samples. Abbreviations: Cyclin dependent kinase 4 (CDK4), Cyclin dependent kinase 6 (CDK6), E2F transcription factor 1 (E2F1), Cyclin D1 (CCND1), Cyclin dependent kinase inhibitor 2A (CDKN2A), Cyclin dependent kinase inhibitor 2B (CDKN2B), Retinoblastoma 1 (RB1), Cyclin E1 (CCNE1), autophagy and beclin-1 regulator 1 (AMBRA1), MYC proto-oncogene (MYC), checkpoint kinase 1 (CHEK1), Family with sequence similarity 122 member A (FAM122A), checkpoint kinase 2 (CHEK2), Ataxia telangiectasia and Rad3-related (ATR), Ataxia-telangiectasia mutated (ATM)

**Supplementary Figure S34**

**Supplementary Fig. S34: Single-cell RNA expression profiles of abemaciclib targets and DDR-associated proteins in publicly available data set (Neftel et al., Cell 2019**^3^**).** UMAP projections display color-coded expression levels across different cell types (legend in the lower right corner); Where gene and protein names differ, the protein name is shown in brackets. Abbreviations: Cyclin dependent kinase 4 (CDK4), Cyclin dependent kinase 6 (CDK6), E2F transcription factor 1 (E2F1), Cyclin D1 (CCND1), Cyclin dependent kinase inhibitor 2A (CDKN2A), Cyclin dependent kinase inhibitor 2B (CDKN2B), Retinoblastoma 1 (RB1), Cyclin E1 (CCNE1), autophagy and beclin-1 regulator 1 (AMBRA1), MYC proto-oncogene (MYC), checkpoint kinase 1 (CHEK1), Family with sequence similarity 122 member A (FAM122A), checkpoint kinase 2 (CHEK2), Ataxia telangiectasia and Rad3-related (ATR), Ataxia-telangiectasia mutated (ATM)

**Supplementary Figure S35**

**Supplementary Fig. S35: Quantitative analysis of single-cell RNA expression profiles of abemaciclib targets and DDR-associated proteins in publicly available data set (Abdelfattah et al., Nat Commun. 2022**^2^**).** Bar plots depict the fraction of cells within the top 10^th^ percentile for each target. Dots are indicative of values from individual samples. Abbreviations: Cyclin dependent kinase 4 (CDK4), Cyclin dependent kinase 6 (CDK6), E2F transcription factor 1 (E2F1), Cyclin D1 (CCND1), Cyclin dependent kinase inhibitor 2A (CDKN2A), Cyclin dependent kinase inhibitor 2B (CDKN2B), autophagy and beclin-1 regulator 1 (AMBRA1), MYC proto-oncogene (MYC), checkpoint kinase 1 (CHEK1), Family with sequence similarity 122 member A (FAM122A), Ataxia telangiectasia and Rad3-related (ATR)

**Supplementary Figure S36**

**Supplementary Fig. S36: Single cell RNA expression profiles of abemaciclib targets and CDK4/6-RB1 associated proteins in publicly available data set (Abdelfattah et al., Nat Commun. 2022**^2^**).** UMAP projections display color-coded expression levels across different cell types (legend in the lower right corner); Where gene and protein names differ, the protein name is shown in brackets. Abbreviations: Cyclin dependent kinase 4 (CDK4), Cyclin dependent kinase 6 (CDK6), E2F transcription factor 1 (E2F1), Cyclin D1 (CCND1), Cyclin dependent kinase inhibitor 2A (CDKN2A), Cyclin dependent kinase inhibitor 2B (CDKN2B), autophagy and beclin-1 regulator 1 (AMBRA1), MYC proto-oncogene (MYC), checkpoint kinase 1 (CHEK1), Family with sequence similarity 122 member A (FAM122A), Ataxia telangiectasia and Rad3-related (ATR)

### Supplementary Table 1: Scoring sheet

| **1** | **general appearance** | |  |
| --- | --- | --- | --- |
| **a** | state of care | clean, shiny, smooth fur | 0 |
|  |  | no fur grooming, dull fur | 1 |
|  |  | no fur grooming, dirty | 2 |
|  |  | no fur grooming, dirty, piloerection | 4 |
| **b** | Eyes | Normal | 0 |
|  |  | subtly sunk in, swollen | 1 |
|  |  | lids closed | 2 |
|  |  | strongly sunken in, lids closed, sticky | 4 |
| **c** | Posture | Normal | 0 |
|  |  | slightly bent | 1 |
|  |  | strongly bent | 2 |
|  |  | strongly bent, paws under the body | 4 |
| **d** | Breathing | Regular | 0 |
|  |  | regular, slightly enhanced | 1 |
|  |  | strongly enhanced | 2 |
|  |  | difficulty breathing, pumping | 4 |
| **e** | behavior/activity | Normal | 0 |
|  |  | slightly changed | 1 |
|  |  | reduced spontaneous-explorative behavior, isolated | 2 |
|  |  | apathic, inactivity | 4 |
| **2** | **nutritional status** | |  |
| **a** | Body condition score | vertebrae and pelvic bones only palpable when slightly pressed | 0 |
|  |  | vertebrae and pelvic bones easily palpable, abdominal retraction detectable from the side | 1 |
|  |  | vertebrae visible, pelvic bones palpable | 2 |
|  |  | vertebrae, pelvic bones and rips visible | 4 |
| **b** | Weight | normal, continuous increase (±5%) | 0 |
|  | calculated from original weight, corrected for expected weight gain of healthy animals | weight loss 5-10% | 1 |
|  |  | weight loss >10 bis <20% | 2 |
|  |  | weight loss max. 20% | **end point** |
| **3** | **experiment associated: tumor growth** | |  |
| **a** | Neurological symptoms: pen test, grid, paw paralysis | None | 0 |
|  |  | slight loss-of-balance, occasionally missed steps, slight paralysis | 1 |
|  |  | Moderate loss-of-balance, every third step missed, moderate paralysis | 2 |
|  |  | Strong loss-of-balance, total inactivity, strong paralysis | **end point** |
| **b** | Grimace Scale | normal | 0 |
|  |  | 1 pain score | 2 |
|  |  | More than 1 pain score | **end point** |
| **end points** | 0-2 points | Normal | |
|  | 3-8 points | daily scoring/weight control, might talk to veterinarians (pain killers, wet food) | |
|  | 9 and more points | **end point** | |
|  | 4 points in experiment associated criteria | **end point** | |
| **exceptional measures** | weight loss 20% | | |
|  | neurological symptoms with strong loss-of-balance, total inactivity and strong paralysis | | |
|  | More than 1 pain score | | |
|  | 1x Score 4 | | |

### Supplementary references

1.	Tarhan L, Bistline J, Chang J, Galloway B, Hanna E, Weitz E. Single Cell Portal: an interactive home for single-cell genomics data. *bioRxiv*. Published online July 17, 2023. doi:10.1101/2023.07.13.548886

2.	Abdelfattah N, Kumar P, Wang C, et al. Single-cell analysis of human glioma and immune cells identifies S100A4 as an immunotherapy target. *Nat Commun*. 2022;13(1). doi:10.1038/S41467-022-28372-Y

3.	Neftel C, Laffy J, Filbin MG, et al. An Integrative Model of Cellular States, Plasticity, and Genetics for Glioblastoma. *Cell*. 2019;178(4):835-849.e21. doi:10.1016/J.CELL.2019.06.024

4.	Jacob F, Salinas RD, Zhang DY, et al. A Patient-Derived Glioblastoma Organoid Model and Biobank Recapitulates Inter- and Intra-tumoral Heterogeneity. *Cell*. 2020;180(1):188-204.e22. doi:10.1016/J.CELL.2019.11.036
